# An Efficient Chemoenzymatic Synthesis of Dihydroartemisinic Aldehyde

**DOI:** 10.1002/anie.201609557

**Published:** 2017-03-13

**Authors:** Melodi Demiray, Xiaoping Tang, Thomas Wirth, Juan A. Faraldos, Rudolf K. Allemann

**Affiliations:** ^1^ School of ChemistryCardiff UniversityMain Building, Park PlaceCardiffCF10 3ATGreat Britain

**Keywords:** artemisinin, chemoenzymatic synthesis, sesquiterpenoids, substrate engineering, synthetic biology

## Abstract

Artemisinin from the plant Artemisia annua is the most potent pharmaceutical for the treatment of malaria. In the plant, the sesquiterpene cyclase amorphadiene synthase, a cytochrome‐dependent CYP450, and an aldehyde reductase convert farnesyl diphosphate (FDP) into dihydroartemisinic aldehyde (DHAAl), which is a key intermediate in the biosynthesis of artemisinin and a semisynthetic precursor for its chemical synthesis. Here, we report a chemoenzymatic process that is able to deliver DHAAl using only the sesquiterpene synthase from a carefully designed hydroxylated FDP derivative. This process, which reverses the natural order of cyclization of FDP and oxidation of the sesquiterpene hydrocarbon, provides a significant improvement in the synthesis of DHAAl and demonstrates the potential of substrate engineering in the terpene synthase mediated synthesis of high‐value natural products.

The sesquiterpenoid endoperoxide artemisinin (**1**) is widely used as a first‐line treatment for malaria in combination therapy.^1^ Although elegant organic syntheses of artemisinin have been published,^2^ the worldwide supply of **1** predominantly relies on extraction from the plant *Artemisia annua*.^3^ The demand for artemisinin is mainly from the developing world, which requires the drug to be produced at low cost. Currently the most efficient way to synthesize artemisinin is to combine biosynthesis with chemical steps. Central to the biosynthesis of **1** (Scheme 1) is the class I sesquiterpene cyclase amorphadiene synthase (ADS), which catalyzes the conversion of (*E*,*E*)‐farnesyl diphosphate (FDP, **2**) into amorpha‐4,11‐diene (**3**). In this complex reaction cascade, two 6‐membered rings, four stereocentres, and two double bonds are formed with exquisite regio‐ and stereochemical control in one step.^4^ Dihydroartemisinic aldehyde (DHAAl, **4**) can be made from amorpha‐4,11‐diene (**3**) either through a three‐step chemical synthesis or by combining a biooxidation with two chemical steps.^5^ Compound **4** can then be converted into **1** chemically or enzymatically in four well‐established steps.2a, 6 It is noteworthy that an elegant semisynthetic pathway has been developed that uses ADS and five other enzymes in yeast to produce artemisinic acid (**5**), which is then converted into dihydroartemisinic acid (**6**) by transition metal‐catalyzed hydrogenation. The pharmaceutical company Sanofi scaled up this process in 2014 but the manufacture was discontinued owing to strong market forces,^7^ thus highlighting the need for ecologically friendly, low‐cost alternatives for the production of artemisinin.

**Scheme 1:**
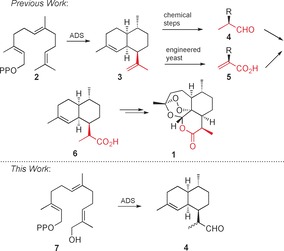
Top: Current routes to artemisinin. Bottom: One‐step ADS‐catalyzed synthesis of dihydroartemisinic aldehyde (DHAAl, **4**) from 12‐hydroxyfarnesyl diphosphate (**7**).

Herein, we report a novel chemoenzymatic process that exploits the substrate promiscuity of ADS to convert the hydroxylated FDP analogue **7** into the synthetic intermediate DHAAl (**4**). In contrast to existing procedures, this process, which reverses the natural order of cyclization of FDP and oxidation of the sesquiterpene hydrocarbon, significantly shortens the synthesis of dihydroartemisinic aldehyde (**4**), in that it uses only one enzyme and requires a single oxidation step that occurs prior to the ADS‐catalyzed cyclization to **4**. The process avoids several redox steps after the cyclization since it bypasses the formation of the intermediate amorphadiene (**3**) altogether (Scheme 1).

Amorphadiene synthase (ADS) catalyzes the Mg^2+^‐dependent conversion of its natural substrate FDP (**2**; see Figure S13 in the Supporting Information) along a complex reaction path that involves isomerization of the 2,3‐double bond from the *E* to the *Z* configuration and 1,6‐cyclization to generate a bisabolyl cation (**8**), followed by a 1,3‐hydride shift from C1 to C7 and 1,10‐cyclization to the amorphyl cation (**9**). Finally, deprotonation at C12 or C13 generates amorpha‐4,11‐diene (**3**; Scheme 2 and Figures S4, S22, S23).^8^

**Scheme 2:**
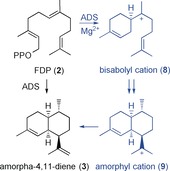
Synthesis of amorphadiene (**3**) from FDP (**2**) in the plant *A. annua*.

Many sesquiterpene synthases display some degree of substrate promiscuity, and are able to convert methylated and fluorinated farnesyl diphosphate analogues into modified terpenoids.^9^, ^10^, ^11^ In this work, we investigated for the first time the potential of a sesquiterpene synthase to accept a hydroxylated FDP analogue as a substrate. In particular, we asked whether ADS11b could convert 12‐hydroxyfarnesyl diphosphate (**7**;^12^ Scheme 1) into dihydroartemisinic aldehyde (**4**) via a 12‐hydroxyamorphyl cation (OH‐**9**; Scheme 3). It is known that α‐hydroxylated carbocations such as OH‐**9** can isomerize under acidic conditions to aldehydes.^13^ The electrophilic nature of terpene synthase chemistry combined with the inherent reactivity of α‐hydroxylated carbocations such as OH‐**9** should therefore lead to aldehyde **4**.

**Scheme 3:**
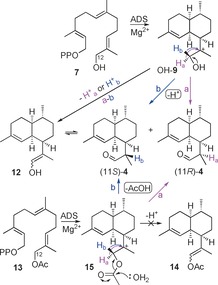
The ADS‐catalyzed synthesis of **4** from 12‐hydroxyfarnesyl diphosphate (**7**) and 12‐acetoxyfarnesyl diphosphate (**13**) via carbocations OH‐**9** and **15**, respectively.

Diphosphate **7** was synthesized in two steps from commercially available (*E,E*)‐farnesyl chloride (**10**; Scheme 4). The short synthesis involved low‐temperature acid‐catalyzed selenium dioxide oxidation of **10** 14 and diphosphorylation of the resulting chloride **11** 15 (Tables S1, S2 and Figures S13–S16). For ^1^H NMR and GC–MS comparison, an authentic sample of aldehyde **4** was prepared from commercially available (11*R*)‐dihydroartemisinic acid (11*R*)‐**6** (Figures S7, S8, S26, S27).6b, 16

**Scheme 4:**
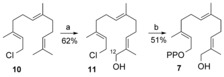
Synthesis of 12‐hydroxyfarnesyl diphosphate (**7**) from commercially available farnesyl chloride **10**. a) SeO_2_ (0.3 equiv), salicylic acid (0.3 equiv), *t*BuOOH (5 equiv), CH_2_Cl_2_, 0 °C. b) (Bu_4_N)_3_HP_2_O_7_ (2 equiv), CH_3_CN, room temperature.

The ^1^H‐NMR spectrum of the purified enzymatic products (Figure 1 and Figures S24, S25) from incubations of **7** with ADS showed the presence of a 3:2 mixture of aldehydes as clearly revealed by their diagnostic NMR signals at *δ*_H_ 9.62 ppm (1 H, d, *J*=3.5 Hz, CHO, major) and 9.57 ppm (1 H, d, *J=*3.5 Hz, CHO, minor). The identity of the minor product was determined to be (11*R*)‐**4** based on ^1^H‐NMR data and comparison with an authentic sample of **4** (Figures S26, S27).17a The structure of the major aldehyde was deduced to be the (11*S*)‐**4** epimer based on its diagnostic ^1^H‐NMR signals at *δ*_H_ 5.27 (s, H2), 1.64 (s, 3‐Me), 1.07 (d, *J=*6.8 Hz, 11‐Me), and 0.87 ppm (d, *J=*6.5 Hz, 7‐Me), which are complementary to those of (11*R*)‐**4** at *δ*_H_ 5.13 (s, H2), 1.64 (s, 3‐Me), 1.06 (d, *J=*6.9 Hz, 11‐Me), and 0.87 ppm (d, *J=*6.5 Hz, 7‐Me; Figure 1). In addition, the epimeric nature of (11*R*)‐**4** and (11*S*)‐**4** is further supported by the epimerization of the corresponding esters that is described later in Scheme 6. Analysis of the NMR sample by GC–MS showed a 7:2 mixture of (11*S*)‐**4** and (11*R*)‐**4**, in addition to approximately 10 % of a minor product (Figure 2) that we tentatively propose to be the enol form (**12**) of aldehyde **4** based on its identical MS spectrum (Figure S3) with that of (11*S*)‐**4**. The MS spectra as well as the GC elution times of (11*R*)‐**4** (faster) and (11*S*)‐**4** are consistent with those reported previously.^17^ The reaction mechanism for the conversion of FDP to amorphadiene (Scheme 2) suggests that enol **12** may initially be formed from carbocation OH‐**9** through deprotonation of either H_a_ or H_b_ (path a‐b in Scheme 3) before **12** equilibrates to the observed mixture of epimers of **4** outside the active site of ADS. However incubations of diphosphate **7** with ADS in D_2_O led to the exclusive production of deuterated (11*S*)‐**4** (Figure S10); no deuterated (11*R*)‐**4** was detected by GC–MS. This observation indicates that only (11*S*)‐**4** can be formed via enol **12**.

**Figure 1:**
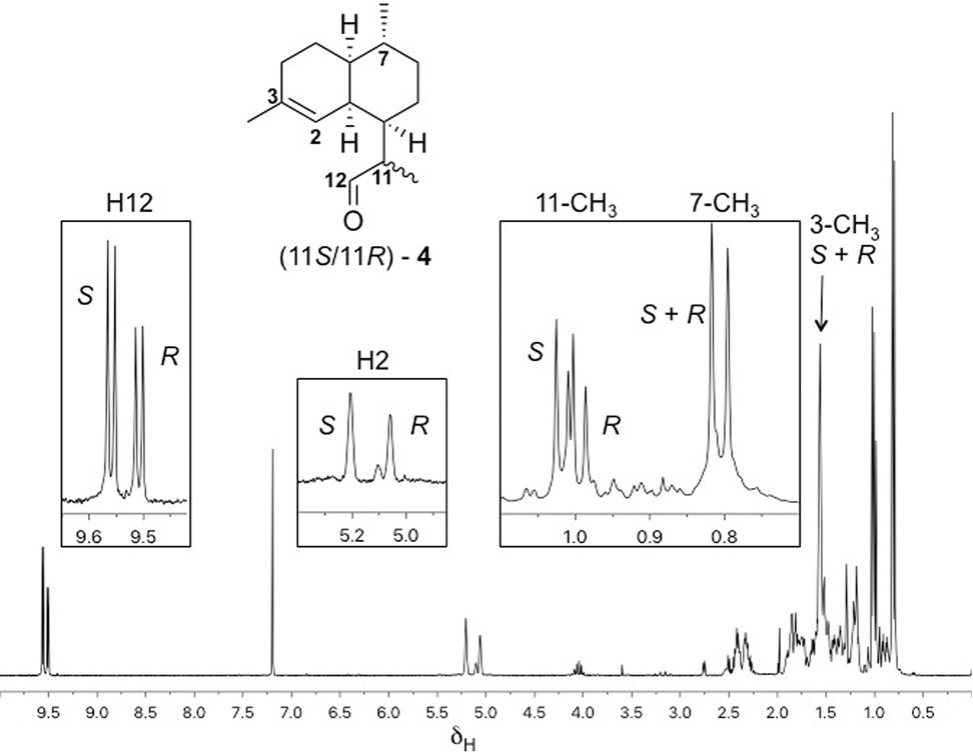
^1^H NMR (500 MHz, CDCl_3_) of the pentane‐extractable products generated by ADS‐catalyzed turnover of **7** after silica‐gel purification, with inserts showing the diagnostic peaks assigned to H12, H2, 11‐CH_3_, and 7‐CH_3_.

**Figure 2:**
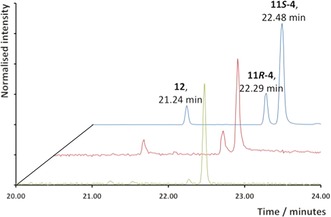
GC chromatogram (TIC) of the pentane‐extractable products from an incubation of diphosphate **7** (blue), **13** (red), and **16** (green) with ADS. GC peaks were assigned as putative enol **12** (21.24 min), and aldehydes (11*R*)‐**4** (22.29 min), and (11*S*)‐**4** (22.48 min).

To further investigate the capability of ADS to act on unnatural substrates, 12‐acetoxyfarnesyl diphosphate (**13**) was prepared in three steps (32 % overall yield) from known 1‐*O*‐THP‐protected 12‐hydroxyfarnesol^14^ (Figures S17, S18) and incubated with ADS. GC–MS analysis revealed that the same three products were obtained as from incubations with diphosphate **7** (Figure 2 and Figure S9). The observation that no vinyl acetate **14** is produced from **13** (Scheme 3) suggests that the enol **12** is not a product of ADS catalysis but is formed exclusively from (11*S*)‐**4** after its release from the active site. Control experiments excluded the presence of ester hydrolase activity in our enzyme preparations (see the Supporting Information). Remarkably, the incubation of 13‐acetoxyfarnesyl diphosphate (**16**, Figure S19) with ADS (Scheme 5) yielded (11*S*)‐**4** (93 %) and enol **12** (7 %; Figure 2 and Figures S5, S6). The absence of significant amounts of the (11*R*)‐epimer of **4** implies that enol **12** does not revert to (11*R*)‐**4** under the reaction conditions (pH≈7.5; Scheme 3). These results strongly suggest that only one pathway operates to yield the observed epimeric mixture of aldehydes **4**. Hence the formation of **4** from **7** (and **13**) most likely proceeds through an oxygen‐assisted non‐stereospecific intramolecular [1,2]‐hydride shift from carbocation OH‐**9** (or **15** for **13**; paths a and b in Scheme 3). Since the stable vinyl acetate **14** is not formed from 13‐acetoxyfarnesyl diphosphate (**16**), deprotonation of **17** via enol **12** to (11*S*)‐**4** appears unlikely. Hence a stereospecific [1,2]‐hydride shift of H_a_ from carbocation **17** most likely accounts for the exclusive formation of (11*S*)‐**4** (path a in Scheme 5) from diphosphate **16**.

**Scheme 5:**
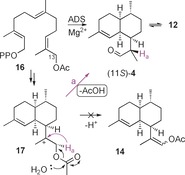
ADS‐catalyzed production of (11*S*)‐**4** from 13‐acetoxyfarnesyl diphosphate (**16**) via carbocation **17**.

To further advance this novel chemoenzymatic approach towards the production of artemisinin, the 2:3 mixture of (11*R*) and (11*S*)‐**4** was oxidized to the corresponding dihydroartemisinic acids (**6**) in 93 % yield (Scheme 6),2b and subsequently converted into the corresponding dihydroartemisinic methyl esters (**18**) in 94 % yield (Figure S11).^18^ Treatment of the esters with LDA under kinetic control resulted in a 1:1 mixture of (11*R*)‐ and (11*S*)‐methyl esters (Figure S28). (11*R*)‐dihydroartemisinic methyl ester is the desired intermediate for the synthesis of artemisinin.^19^

**Scheme 6:**
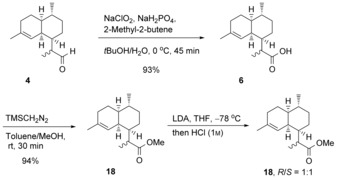
Synthesis of dihydroartemisinic acid methyl ester (**18**).

In conclusion, we have developed an efficient chemoenzymatic route to dihydroartemisinic aldehyde (**4**), a major intermediate in the production of artemisinin (**1**), the most important drug for the treatment of malaria. We have for the first time shown that hydroxylated FDP analogues can be accepted as substrates by sesquiterpene synthases. Hence our work offers a novel “reversed biosynthetic” approach for the synthesis of functionally diversified hydroxylated terpenoids. The chemical synthesis of such products is often difficult owing to the need to make several small rings with high stereo‐ and regiocontrol. The relatively high substrate promiscuity and the templating effect of the active site allow terpene synthases to chaperone unnatural substrates along well‐defined reaction paths to specific products with high fidelity. This design offers a promising approach for the production of high‐value terpenoids and terpene alkaloids11a that is complementary to conventional synthetic and biosynthetic procedures.

## Conflict of interest

The authors declare no conflict of interest.

## Supporting information

As a service to our authors and readers, this journal provides supporting information supplied by the authors. Such materials are peer reviewed and may be re‐organized for online delivery, but are not copy‐edited or typeset. Technical support issues arising from supporting information (other than missing files) should be addressed to the authors.

Supplementary
