# Supplementary material for: An Efficient Chemoenzymatic Synthesis of Dihydroartemisinic Aldehyde
Source: Angew Chem Int Ed Engl. 2017 Mar 13;56(15):4347–50. doi: 10.1002/anie.201609557 (PMC5396139; doi:10.1002/anie.201609557)
Supplement: Supplementary file 1 — Supplementary [file ANIE-56-4347-s001.pdf]

Angewandte Chemie
Eine Zeitschrift der Gesellschaft Deutscher Chemiker

Supporting Information

An Efficient Chemoenzymatic Synthesis of Dihydroartemisinic Aldehyde

*Melodi Demiray, Xiaoping Tang, Thomas Wirth, Juan A. Faraldos, and Rudolf K. Allemann**

anie_201609557_sm_miscellaneous_information.pdf

Supporting information for

An efficient chemoenzymatic synthesis of dihydroartemisinic aldehyde

Melodi Demiray, Xiaoping Tang, Thomas Wirth, Juan A. Faraldos, and Rudolf K. Allemann*

Table of contents

1. General methods and materials 1
2. Synthetic procedures 2
3. Preparation of amorphadiene synthase (ADS) 15
4. Steady state kinetics 16
5. Optimised synthesis of 12-hydroxyfarnesyl diphosphate (**7**) 18
6. Optimization of the catalytic turnover of **7** to dihydroartemisinic aldehyde (**4**) 18
7. Enzymatic Incubations 19
8. GC-MS 21
9. NMR spectra 29
10. References 46

1. General methods and materials

For synthetic procedures, all chemicals and solvents were obtained from commercial vendors and used without further purification unless otherwise noted. Anhydrous tetrahydrofuran (THF), diethyl ether, toluene and acetonitrile were obtained from a MBraun SPS800 solvent purification system. Dichloromethane and triethylamine were distilled from calcium hydride and potassium hydroxy under nitrogen, respectively**.** TLC visualisations were performed with 4.2% ammonium molybdate and 0.2% ceric sulfate in 5% H_2SO_4 or UV light. Flash chromatography was performed according to the method of Still.[1]

^{1}H- and ^{13}C-NMR spectra were measured on a Bruker Avance 500 NMR spectrometer or a Bruker Fourier 300 NMR spectrometer and are reported as chemical shifts in parts per million downfield from tetramethylsilane; multiplicity (s = singlet, d = doublet, t = triplet, q = quartet, m = multiplet), coupling (to the nearest 0.5 Hz) and assignment, respectively. GC-MS analysis of incubation products was performed on a Hewlett Packard 6890 GC apparatus fitted with a J&W scientific DB-5MS column (30 m x 0.25 mm internal diameter) and a Micromass GCT Premiere detecting in the range *m/z* 50-800 in the EI^+ mode with scanning once a second with a scan time of 0.9 s. Method: The program used an injection port temperature of 100 °C; split ratio 5:1; initial temperature 50 °C, hold 1 min, ramp of 4 °C/min to 150 °C hold 15 min, ramp of 20 °C/min to 250 °C hold 3 min. GC-FID analyses of incubation products were measured using an Agilent 7890A GC system with a manual injector and a SUPELCO Aztec CHIRALDEXTM B-DM silica capillary column. Carrier gas was helium (flow rate: 1 mL min^{-1}, split ratio 20:1) and the method used was isothermal, the oven temperature was held at 150 °C for 30 minutes. High-resolution ES^- mass spectra were measured on a Micromass LCT premiere XE spectrometer fitted with a Waters 1525 Micro binary HPLC pump. The purity of final compounds was judged to be > 95% by TLC and/or GC analyses and NMR spectra analysis.

2. Synthetic procedures

Farnesyl diphosphate was synthesised as described by Davisson *et al.* [2]

Farnesal was synthesised as described by Masuda *et al.* [3]

2.1. Preparation of 12-hydroxyfarnesyl diphosphate (7) and 12-acetoxyfarnesyl diphosphate (13).

2-(((2*E*, 6*E*)-3, 7, 11-trimethyl dodeca-2, 6, 10-trien-1-yl) oxy) tetrahydro-2*H*-pyran

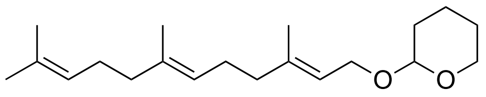

A solution of farnesol (5.0 g, 22.7 mmol) in DCM (25 mL) was added to 3,4-dihydropyran (8.1 mL, 88.8 mmol) and left to stir for 10 min. Pyridinium *p*-toluenesulfonate (0.6 g, 2.4 mmol) was added and the mixture was stirred for 1 h whereupon TLC analysis (8:2, hexane: ethyl acetate) showed full consumption of starting material. DCM was removed and the residual crude was dissolved in diethyl ether (50 mL). The organic solution was washed with saturated aqueous sodium bicarbonate solution (3 x 20 mL) and brine (20 mL), before being dried over anhydrous magnesium sulfate, filtered under gravity and concentrated under reduced pressure to give the title compound as a colourless oil (6.5 g, 93%). The product was judged by ^{1}H-NMR spectroscopy to be sufficiently pure to carry forward without further purification.

^{1}H-NMR (300 MHz, $CDCl_3$): δ 5.36 (1 H, t, $J_{H,H}$ = 6.5, $C\underline{H}CH_2O$), 5.10-5.13 (2 H, m, 2 x $CC\underline{H}CH_2CH_2$), 4.63 (1 H, t, $J_{H,H}$ = 4.0, $OC\underline{H}O$), 4.24 (1 H, dd, $J_{H,H}$= 12.0 and 6.5, $CCHC\underline{H_A}O$), 4.06 (1 H, dd, $J_{H,H}$= 12.0 and 7.5, $CCHC\underline{H_B}O$), 3.93-3.47 (2 H, m, $OC\underline{H_2}CH_2$), 2.16-1.94 (8 H, m, 2 x $CCHC\underline{H_2}C\underline{H_2}$), 1.89-1.50 (6 H, m, $OCHC\underline{H_2}C\underline{H_2}C\underline{H_2}$), 1.68 and 1.60 (2 x 6 H, 2 x s, 4 x $CC\underline{H_3}$). **^{13}C-NMR** (75 MHz, $CDCl_3$): δ 140.5, 135.4 and 131.5 (3 x $CH_3\underline{C}CH$), 124.5, 124.1 and 120.7 (3 x $CH_3C\underline{C}H$), 97.9 ($O\underline{C}HO$), 63.8 and 62.5 (2 x $CHO\underline{C}H_2$), 39.9 and 39.8 (2 x $CH_3C\underline{C}H_2$), 30.9 ($OCH\underline{C}H_2$), 26.9 and 26.47 (2 x $CCH\underline{C}H_2CH_2$), 25.90 ($OCH_2\underline{C}H_2$), 25.7 ($OCH_2CH_2\underline{C}H_2$), 19.8, 17.9, 16.6 and 16.2 (4 x $C\underline{C}H3$). **HRMS** (ES^+): calculated for $C_{20}H_{34}O_2[Na]^+$: 329.2457, found: 329.2463.

(2*E*, 6*E*, 10*E*)-2, 6, 10-trimethyl-12-((tetrahydro-2H-pyran-2-yl) oxy) dodeca-2, 6, 10-trien-1-ol

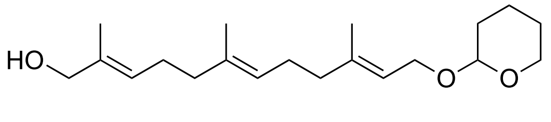

Selenium dioxide (109 mg, 1.0 mmol), salicylic acid (135 mg, 1.0 mmol), and *tert*-butyl hydroperoxide (1.6 mL, 16.3 mmol) were stirred in dichloromethane (20 mL) for 30 min at room temperature. The temperature was reduced to 0 °C before the addition of the THP protected farnesol (1 g, 3.3 mmol). The reaction was left to stir for 24 h whilst remaining between 0 – 4 °C. DCM was removed using reduced pressure and *tert*-butyl hydroperoxide was removed by co-evaporation with toluene (2 x 20 mL) under reduced pressure. The crude residue was dissolved in diethyl ether (20 mL) and washed with saturated aqueous sodium bicarbonate solution (50 mL). The biphasic layers were separated and

the aqueous layer was further washed with diethyl ether (5 x 20 mL). The combined organic layers were washed with brine (20 mL) and dried over anhydrous magnesium sulfate before being filtered under gravity. The solvent was removed under reduced pressure and the crude oil was purified by flash chromatography on silica gel (9:1, hexane: ethyl acetate) to yield the product as a colourless oil (0.5 g, 48%).

^{1}H-NMR (300 MHz, $CDCl_3$): δ 5.41-5.33 (2 H, m, 2 x $CC\underline{H}CH_2$), 5.11 (1 H, t, $J_{H,H}$ = 6.5, $CC\underline{H}CH_2$), 4.63 (1 H, t, $J_{H,H}$ = 4.0, $OC\underline{H}O$), 4.23 (1 H, dd, $J_{H,H}$ = 12.0 and 6.5, $CCHC\underline{H_A}O$), 4.01 (1 H, dd, $J_{H,H}$ = 12.0 and 7.5, $CCHC\underline{H_B}O$), 3.99 (2 H, s, $OHC\underline{H_2}$), 3.93-3.48 (2 H, m, $OC\underline{H_2}CH_2$), 2.20-1.99 (8 H, m, 2 x $CCHC\underline{H_2}C\underline{H_2}$), 1.75-1.50 (6 H, m, $CHC\underline{H_2}C\underline{H_2}C\underline{H_2}$), 1.68, 1.66 and 1.60 (3 x 3 H, 3 x s, 3 x $CC\underline{H_3}$). **^{13}C NMR** (75 MHz, $CDCl_3$): δ 140.2, 135.0 and 135.0 (3 x $CH_3\underline{C}CH$), 126.1, 124.5 and 121.0 (3 x $CH_3\underline{C}CH$), 97.9 ($O\underline{C}HO$), 69.1 ($OH\underline{C}H_2$), 63.8 and 62.5 (2 x $CHO\underline{C}H_2$), 39.7 and 39.5 (2 x $C\underline{C}H_2CH_2$), 30.9 ($OC\underline{C}H_2$), 26.4 and 26.3 (2 x $CCH_2\underline{C}H_2$), 25.7 ($OCH_2\underline{C}H_2CH_2$), 19.8 ($OCH_2CH_2\underline{C}H_2$), 16.6, 16.2 and 13.8 ($C\underline{C}H_3$). **HRMS** ($ES^+$): calculated for $C_{20}H_{34}O_3[Na]^+$: 345.2406, found: 345.2415.

(2*E*, 6*E*, 10*E*)-2, 6, 10-trimethyl-12-((tetrahydro-2H-pyran-2-yl) oxy) dodeca-2, 6, 10-trien-1-yl acetate

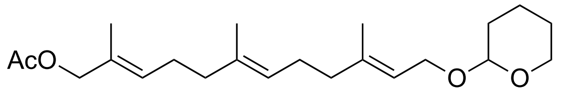

To a stirred solution of the above alcohol (1.3 g, 4.1 mmol) in dichloromethane (20 mL), acetic anhydride (1.5 mL, 16.1 mmol) and pyridine (1.3 mL, 16.1 mmol) were added. The reaction was left to stir at room temperature for 24 h and was quenched with saturated aqueous sodium bicarbonate solution (20 mL). The biphasic layers were separated and the aqueous layer was washed with diethyl ether (4 x 20 mL). The combined organic layers were washed with brine (5 x 20 mL) and dried over anhydrous magnesium sulfate before being filtered under gravity. The solvent was removed under reduced pressure and the crude oil was purified by flash chromatography on silica gel (8:1, hexane: ethyl acetate) to give the corresponding acetate as a colourless oil (1.4 g, 95%).

^{1}H-NMR (300 MHz, $CDCl_3$): δ 5.44 (1 H, t, $J_{H,H}$ = 6.5, $CC\underline{H}CH_2$), 5.36 (1 H, t, $J_{H,H}$ = 7.0, $CC\underline{H}CH_2$), 5.11 (1 H, t, $J_{H,H}$ = 7.0, $CC\underline{H}CH_2$), 4.62 (1 H, t, $J_{H,H}$ = 4.0, $OC\underline{H}O$), 4.44 (2 H, s, $CC\underline{H_2}O$), 4.24 (1 H, dd, $J_{H,H}$ = 12.0 and 6.5, $CCHC\underline{H_A}O$), 4.02 (1 H, dd, $J_{H,H}$ = 12.0 and 7.5, $CCHC\underline{H_B}O$), 3.93-3.47 (2 H, m, $OC\underline{H_2}CH_2$), 2.20-1.98 (8 H, m, 2 x $CCHC\underline{H_2}C\underline{H_2}$), 2.07 (3 H, s, $OCC\underline{H_3}$), 1.75-1.49 (6 H, m, $CHC\underline{H_2}C\underline{H_2}C\underline{H_2}$), 1.68, 1.65 and 1.60 (3 x 3 H, 3 x s, 3 x $CC\underline{H_3}$). **^{13}C-NMR** (75 MHz, $CDCl_3$): δ 171.1 ($CH_3\underline{C}O$), 140.2, 134.7 and 130.0 (3 x $CH_3\underline{C}CH$), 129.6, 124.3 and 120.6 (3 x $CH_3C\underline{C}H$), 97.8 ($O\underline{C}HO$), 70.5 ($C\underline{C}H_2O$), 63.8 and 62.5 (2 x $CHO\underline{C}H_2$), 39.7 and 39.2 (2 x $C\underline{C}H_2CH_2$), 30.9 ($OC\underline{C}H_3$), 26.5 and 26.5 (2 x $CCH_2\underline{C}H_2$), 25.7 ($OCH_2\underline{C}H_2CH_2$), 19.8 ($OCH_2CH_2\underline{C}H_2$), 16.6, 16.2 and 14.1 (3 x $C\underline{C}H_3$). **HRMS** (AP^+): calculated for $C_{22}H_{36}O_4[Na]^+$: 387.2511, found: 387.2501.

(2*E*, 6*E*, 10*E*)-12-hydroxy-2, 6, 10-trimethyl dodeca-2, 6, 10-trien-1-yl acetate

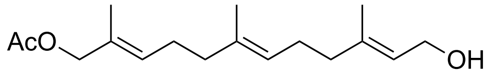

The acetate (930 mg, 2.5 mmol) and *p*-toluenesulfonic acid (47 mg, 247 μmol) were mixed in methanol (20 mL) and stirred at room temperature for 1 h until TLC analysis (8:2, hexane: ethyl acetate) confirmed complete consumption of starting material. The solvent was removed under reduced pressure and the residual oil was dissolved with diethyl ether (20 mL) and washed with saturated aqueous sodium bicarbonate solution (3 x 10 mL) and brine (10 mL). The resulting solution was dried over anhydrous magnesium sulfate, filtered under gravity and concentrated under reduced pressure to give the title compound as a colourless oil (690 mg, 97%). The product was judged by ^{1}H NMR spectroscopy to be sufficiently pure to be carried forward without further purification.

^{1}H-NMR (300 MHz, $CDCl_3$): δ 5.46-5.39 (2 H, m, 2 x CC$\underline{H}$), 5.11 (1 H, t, $J_{H,H}$ = 6.5, CC$\underline{H}$CH$_2$), 4.44 (2 H, s, OC$\underline{H_2}$C), 4.15 (2 H, d, $J_{H,H}$ = 7.0, C$\underline{H_2}$OH), 2.14-2.00 (8 H, m, 2 x CCHC$\underline{H_2}$C$\underline{H_2}$), 2.07 (3 H, s, OCC$\underline{H_3}$), 1.68, 1.65 and 1.60 (3 x 3 H, 3 x s, 3 x CC$\underline{H_3}$). **^{13}C-NMR** (75 MHz, $CDCl_3$): δ 171.3 (CH$_3$$\underline{C}$O), 139.8, 135.0 and 130.1 (3 x CH$_3$$\underline{C}$CH), 129.8, 124.4 and 123.6 (3 x CH$_3$C$\underline{C}$H), 70.6 (C$\underline{C}$H$_2$O), 59.6 ($\underline{C}$H$_2$OH), 39.6 and 39.2 (2 x C$\underline{C}$H$_2$CH$_2$), 26.5 and 26.4 (2 x CCH$_2$$\underline{C}H_2$), 21.2 (OC$\underline{C}H_3$), 16.5, 16.1 and 14.2 (3 x CHCC$\underline{H_3}$). **HRMS** (ES$^+$): calculated for $C_{17}H_{28}O_3[Na]^+$: 303.1936, found: 303.1945.

12-Acetoxyfarnesyl diphosphate (13)

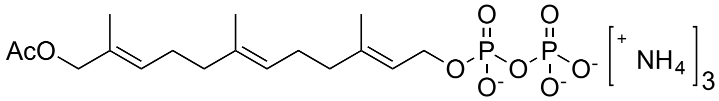

To a cold suspension (0 °C) of 12-acetoxyfarnesol (900 mg, 3.2 mmol), lithium chloride (545 mg, 12.9 mmol) and *s*-collidine (2.5 mL, 19.3 mmol) in anhydrous DMF (10 mL) methanesulfonyl chloride (0.5 mL, 6.4 mmol) was added under nitrogen. The solution was stirred for 3 h, poured into cold water (50 mL) and extracted with diethyl ether (4 x 20 mL). The combined organic extracts were washed with saturated aqueous copper sulfate solution (3 x 20 mL), water (2 x 20 mL) and saturated aqueous ammonium bicarbonate solution (2 x 20 mL), dried over anhydrous magnesium sulfate, filtered under gravity and concentrated under reduced pressure to give the crude chloride intermediate.

To a stirred solution of $(Bu_4N)_3HP_2O_7$ (5.8 g, 6.4 mmol) in anhydrous acetonitrile (10 mL) under N_2 was added to a solution of the crude chloride in acetonitrile (5 mL). This was left for 24 h at room temperature. The solvent was removed under reduced pressure and the resulting oil was dissolved in a minimum amount of ion-exchange buffer (25 mM ammonium bicarbonate solution containing 2% isopropanol).

The crude compound in buffer was then eluted through the NH_4^+-form cation exchange column with additional ion exchange buffer. Fractions that contained the diphosphate, TLC (6:3:1 isopropanol: water: ammonium hydroxide) were collected and lyophilized. The resulting dry powder was purified by HPLC to give **13** as a white solid (0.6 g, 35%) after lyophilization.

^{1}H-NMR (500 MHz, D_2O): δ 5.51-5.46 (2 H, m, 2 x CC$\underline{H}$), 5.22 (1 H, t, $J_{H,H}$ = 6.5, CC$\underline{H}$), 4.50 (2 H, s, CC$\underline{H_2}$O), 4.48 (2 H, t, $J_{H,H}$ = 6.5, CHC$\underline{H_2}$O), 2.25-2.03 (8 H, m, 2 x CCHC$\underline{H_2}$C$\underline{H_2}$), 2.10 (3 H, s,

OCC$\underline{H}_3$), 1.73, 1.65 and 1.63 (3 x 3 H, 3 x s, 3 x CHCC$\underline{H}_3$). **^{31}P-NMR** (121 MHz, D_2O): δ -10.33 (d, *J* = 21.5), -6.57 (d, *J* = 21.5) **HRMS** (ES^-): calculated for $C_{17}H_{27}O_9P_2[2H]^-$: 439.1287, found: 439.1290.

12-Hydroxyfarnesyl diphosphate (7)

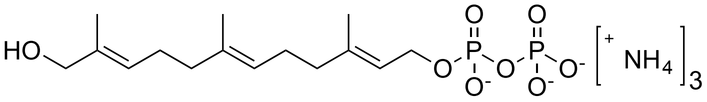

To a stirred solution of **13** (0.3 g, 0.6 mmol) in deuterated water (10 mL) ammonium hydroxide (28% NH_3 in H_2O, 2 mL) was added and left to stir for 24 h. The following day ^{1}H NMR analysis continued to show the presence of the acetate group. Additional conc. ammonium hydroxide (28% NH_3 in H_2O, 2 mL) was added and left for another 24 h. The following day the deuterated water and ammonium hydroxide was lyophilized to give **7** as a white solid (290 mg, 97%).

M.p. 132-136 °C **^{1}H-NMR** (500 MHz, D_2O): δ 5.41-5.33 (2 H, m, 2 x CC$\underline{H}$), 5.15 (1 H, t, $J_{H,H}$ = 6.5, CC$\underline{H}$), 4.40 (2 H, t, $J_{H,H}$ = 6.5, CHC$\underline{H}_2$O), 3.88 (2 H, s, CC$\underline{H}_2$OH), 2.16-1.96 (8 H, m, 2 x CCHC$\underline{H}_2$C$\underline{H}_2$), 1.65, 1.57 and 1.56 (3 x 3 H, 3 x s, 3 x CC$\underline{H}_3$). **^{31}P-NMR** (121 MHz, D_2O): δ -7.93 (d, *J* = 17.0 Hz), -7.53 (m). **HRMS** (ES^-): calculated for $C_{15}H_{25}O_8P_2[H][Na]^-$: 419.1001, found: 419.0983.

2.2 Preparation of 12-hydroxyfarnesyl diphosphate (12-OH FDP, 7) under optimised conditions (See Tables S1 and S2 on page S17)

12-Hydroxyfarnesyl chloride (11)

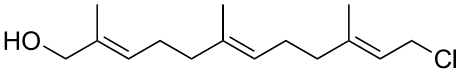

Selenium dioxide (66.0 mg, 0.6 mmol), salicylic acid (82.0 mg, 0.6 mmol) and 90% *tert*-butyl hydroperoxide (1 mL, 10 mmol) were dissolved in dichloromethane (10 mL) and left to stir for 30 min at room temperature. The reaction mixture was then cooled to 0 ºC after which farnesyl chloride **10** (450 mg, 2 mmol) in dichloromethane (5 mL) was added and left to stir for 24 h at 0 ºC. The reaction was quenched with saturated sodium bicarbonate solution (20 mL). Dichloromethane was removed under reduced pressure. The *tert*-butyl hydroperoxide was removed by repeated addition of toluene and co-evaporation under reduced pressure. The resulting crude mixture was diluted with diethyl ether (20 mL) and the aqueous layer was separated. This was extratced with additional diethyl ether (3 x 20 mL). The organic extracts were combined, washed with brine (10 mL) and dried over anhydrous sodium sulphate. After filtration, the solvent was removed under reduced pressure. The crude oil was purified by flash chromatography on silica gel (9.5: 0.5, hexane: ethyl acetate) to give starting material (75 mg) and the title compound as a yellow oil (245 mg, 61.5%). In addition to 12-hydroxy farnesyl chloride, 8-hydroxy farnesyl chloride (79 mg, 19.2%) and 12-aldehyde farnesyl chloride (73 mg, 18.4%) were also produced as side products.

12-Hydroxy farnesyl chloride: ^{1}**H-NMR** (300 MHz, $CDCl_3$): δ (ppm) 5.47-5.36 (2 H, m, 2 x $C\underline{H}$), 5.10 (1 H, t, $J_{H,H}$ = 6.0,$C\underline{H}$), 4.10 (2 H, d, $J_{H,H}$ = 8.0, $C\underline{H_2}Cl$), 3.99 (2 H, s, $C\underline{H_2}OH$), 2.16-1.99 (8 H, m, 2 x $CCHC\underline{H_2}C\underline{H_2}$), 1.73, 1.66 and 1.60 (3 x 3 H, 3 x s, 3 x $CC\underline{H_3}$). ^{13}**C-NMR** (75 MHz, $CDCl_3$) 142.7, 135.3, 134.7, 126.0, 123.7, 120.3, 69.0, 41.2, 39.4, 39.3, 26.2, 26.1, 16.1, 16.0, 13.7. **HRMS** (ES^+): calcd for $C_{15}H_{25}OCl[Na]^+$: 279.1483, found: 279.1492.

8-Hydroxy farnesyl chloride: ^{1}**H-NMR** (300 MHz, $CDCl_3$): δ (ppm) 5.44 (1 H, t, $J_{H,H}$ = 8.0, $C{=}C\underline{H}$), 5.36 (1 H, t, $J_{H,H}$ = 6.5, $C{=}C\underline{H}$), 5.08 (1 H, t, $J_{H,H}$ = 8.0, $C{=}C\underline{H}$), 4.10 (2 H, d, $J_{H,H}$ = 8.0, $C\underline{H_2}Cl$), 3.97 (1 H, t, $J_{H,H}$ = 7.5, $C\underline{H}OH$), 2.33-2.05 (7 H, m, $C{=}CHC\underline{H_2}C\underline{H_2}$ and $C{=}CHC\underline{H}OHC\underline{H_2}$), 1.73, 1.64 and 1.62 (3 x 3 H, 3 x s, 3 x $CC\underline{H_3}$).

12-Aldehyde farnesyl chloride: ^{1}**H-NMR** (300 MHz, $CDCl_3$): δ (ppm) 9.38 (1 H, s, $C\underline{H}O$), 6.46 (1 H, t, $J_{H,H}$ = 8.0, $OCHC{=}C\underline{H}$), 5.43 (1 H, t, $J_{H,H}$ = 8.0,$C\underline{H}$), 5.13 (1 H, t, $J_{H,H}$ = 6.5,$C\underline{H}$), 4.09 (2 H, d, $J_{H,H}$ = 8.0, $C\underline{H_2}Cl$), 2.49-2.41 (2 H, m, $CCHC\underline{H_2}CH_2$), 2.19-2.06 (6 H, m, $CCHCH_2C\underline{H_2}$ and $CCHC\underline{H_2}C\underline{H_2}$), 1.73, 1.72 and 1.62 (3 x 3 H, 3 x s, 3 x $CC\underline{H_3}$). ^{13}**C-NMR** (75 MHz, $CDCl_3$) 195.5, 154.7, 142.6, 139.5, 134.2, 126.0, 124.9, 120.6, 41.3, 39.4, 38.1, 27.6, 26.2, 18.6, 16.3, 16.2, 13.7, 9.4.

Tris-(tetrabutyl ammonium) 12-hydroxyfarnesyl diphosphate (7)

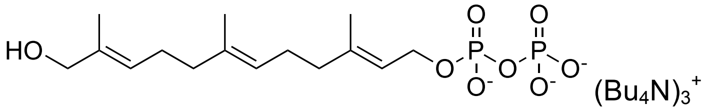

Chloride **11** (0.24 g, 0.94 mmol) was dissolved in anhydrous acetonitrile with powdered molecular sieves (3Å, 1 g) under argon and treated with $(Bu_4N)_3HP_2O_7$ (1.70 g, 1.88 mmol). The reaction was stirred for 48 h. The reaction mixture was directly purified by flash chromatography on silica gel (6: 2.5: 0.5, isopropanol: ammonium hydroxide: water) to give **7** as a colourless oil (0.54 g, 51%).

^{1}**H-NMR** (300 MHz, $CDCl_3$): δ (ppm) 5.37 (1 H, t, J = 6.5, $C\underline{H}$), 5.33 (1 H, t, $J_{H,H}$ = 7.0, $C\underline{H}$), 5.05 (1 H, t, $J_{H,H}$ = 7.0, $C\underline{H}$), 4.49 (2 H, t, $J_{H,H}$ = 6.0, $C\underline{H_2}OP$), 3.97 (2 H, s, $C\underline{H_2}OH$), 3.32 (24 H, m, 12 x $NC\underline{H_2}CH_2CH_2CH_3$), 2.11-2.00 (8 H, m, 2 x $CCHC\underline{H_2}C\underline{H_2}$), 1.67-1.58 (24 H, m, 12 x $NCH_2C\underline{H_2}CH_2CH_3$), 1.62, 1.59 and 1.55 ((3 x 3 H, 3 x s, 3 x $CHCC\underline{H_3}$), 1.48-1.41 (12 x $NCH_2CH_2C\underline{H_2}CH_3$), 0.99-0.96 (36 H, t, $J_{H,H}$ = 7.5, 12 x $NCH_2CH_2CH_2C\underline{H_3}$). ^{31}**P-NMR** (202 MHz, $CDCl_3$), -7.50 (d, J= 17.0), -7.87 (d, J= 17.0). **HRMS** (ES^-): calculated for $C_{15}H_{26}O_8P_2[2H]^-$: 419.0983, found: 419.1001.

2.3 Preparation of (11*R*)-dihydroartemisinic aldehyde (11*R*-4) from (11*R*)-dihydroartemisinic acid (11*R*-6)

(11*R*)-dihydroartemisinic methyl ester

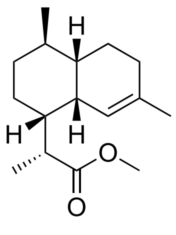

Dimethyl formamide (10 μL) was added to (11*R*)-dihydroartemisinic acid (25 mg, 0.10 mmol) in toluene (2 mL). The reaction was stirred and maintained at 0 °C while oxalyl chloride (9.4 mL, 0.11mmol) was added dropwise. The solution was left to stir for 30 min before a mixture of triethylamine (100 μL) and methanol (900 μL) was added dropwise over 10 min. The mixture was stirred for 1 hr and then the excess methanol was removed under reduced pressure. The toluene suspension was washed with water (2 x 5 mL), dried over anhydrous sodium sulfate, filtered under gravity and concentrated under reduced pressure. The crude oil was purified by flash chromatography on silica gel (9:1, hexane: ethyl acetate) to give the title compound as a colourless oil (14 mg, 78%).
^{1}H-NMR (500 MHz, $CDCl_3$): δ 5.12 (1 H, bs, $CH_3C{=}C\underline{H}$), 3.67 (3 H, s, $C\underline{H_3}OC$), 2.50 (2 H, m, $OCC\underline{H}CHC\underline{H}$), 1.97-1.78 (3 H, m, $CH_3CC\underline{H_2}C\underline{H_b}$), 1.63 (3 H, s, $CH{=}CC\underline{H_3}$), 1.63-1.55 (3 H, m, $CH_3CCH_2C\underline{H_a}$ and $OCCHC\underline{H}$ and $CH_3CHC\underline{H_b}$), 1.41 (1 H, m, $CH_3C\underline{H}CH_2$), 1.26 (2 H, m, $OCCHCHC\underline{H_a}$ and $CH{=}CCH_2CH_2C\underline{H}$), 1.13 (3 H, d, $J_{H,H}$ = 7.0, $CHC\underline{H_3}$), 1.09-0.92 (2 H, m, $CH3CHC\underline{H_a}C\underline{H_b}$), 0.86 (3 H, d, $J_{H,H}$ = 6.5, $CHC\underline{H_3}$). **^{13}C-NMR** (125 MHz, $CDCl_3$): δ 178.2 ($O\underline{C}O$), 136.1 ($CH{=}\underline{C}CH_3$), 119.7 ($\underline{C}H{=}CCH_3$), 51.6 ($CO\underline{C}H_3$), 44.2 ($\underline{C}HCHCO$), 43.3 ($CH\underline{C}HCO$), 41.9 ($\underline{C}HCH_2CH_2C{=}CH$), 36.6 ($\underline{C}H{=}CCH_3$), 35.5 ($CH_3CH\underline{C}H_2CH_2$), 27.9 ($CH_3\underline{C}HCH_2CH_2$), 27.7 ($CH_3CHCH_2\underline{C}H_2$), 26.8 ($CH_2\underline{C}H_2C{=}CH$), 26.0 ($\underline{C}H_2CH_2C{=}CH$), 24.0 ($\underline{C}H_3CHCH_2$), 19.9 ($CH{=}C\underline{C}H_3$), 15.3 ($\underline{C}H_3CHCO$).

(11*R*)-dihydroartemisinic aldehyde (11*R*-4)

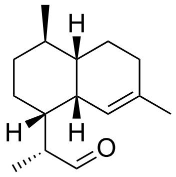

(11*R*)-dihydroartemisinic methyl ester (11 mg, 44 μmol) was cooled to -78 °C and stirred in anhydrous THF (1 mL). Diisobutylaluminium hydride (1 M in toluene, 44 μL, 44 μmol) was added drop-wise and the reaction was left to stir for 30 min. The reaction was quenched with saturated aqueous sodium bicarbonate solution (5 mL) and left to stir for 1 h. The layers were separated and the aqueous layer was washed with diethyl ether (3 x 5 mL). Combined organic extracts were washed with brine (10 mL) and dried over anhydrous sodium sulfate before being filtered under gravity and concentrated under reduced pressure. The crude oil was purified by flash chromatography on silica gel (19:1, hexane: ethyl acetate) to the title compound as a colourless oil (3 mg, 31%).

^{1}H-NMR (500 MHz, $CDCl_3$): δ (ppm) 9.57 (1 H, d, $J_{H,H}$ = 3.5, $C\underline{H}O$), 5.12 (1 H, bs, $CH_3C{=}C\underline{H}$), 2.48 (1 H, m, $C{=}CHC\underline{H}$), 2.37 (1 H, m, $OCHC\underline{H}$), 1.98-1.79 (3 H, m, $CH_3CC\underline{H_2}C\underline{H_b}$), 1.64 (3 H, s, $CC\underline{H_3}$), 1.63-1.56 (3 H, m, $CH_3CCH_2C\underline{H_a}$ and $OCHCHC\underline{H}$ and $CH_3CHC\underline{H_b}$), 1.41 (2 H, m, $CH_3C\underline{H}CH_2$ and $OCHCHCHC\underline{H_a}$), 1.29 (1 H, m, $CH{=}CCH_2CH_2C\underline{H}$), 1.13 (1 H, dd, $J_{H,H}$ = 13.0 and 3.0, $OCHCHCHC\underline{H_b}$), 1.07 (3 H, d, $J_{H,H}$ = 7.0, $OCHCHC\underline{H_3}$), 0.96 (1 H, ddd, $J_{H,H}$ = 24.5, 13.0 and 3.0, $CH_3CHC\underline{H_2}$), 0.88 (3 H, d, $J_{H,H}$ = 6.5, $CHC\underline{H_3}$). **^{13}C-NMR** (125 MHz, $CDCl_3$): δ 206.3 ($\underline{C}HO$), 136.3 ($CH{=}\underline{C}CH_3$), 119.8 ($\underline{C}H{=}CCH_3$), 48.7 ($OCH\underline{C}H$), 42.1 ($CH_3CCH_2CH_2\underline{C}H$), 41.7 ($OCHCH\underline{C}H$), 36.8

(C=CH$\underline{C}$H), 35.5 ($CH_3CH\underline{C}H_2CH_2$), 27.9 ($CH_3\underline{C}HCH_2CH_2$), 27.6 ($CH_3CHCH_2\underline{C}H_2$), 26.8 ($CH_2\underline{C}H_2C$=CH), 26.0 ($\underline{C}H_2CH_2C$=CH), 24.0 ($\underline{C}H_3C$=CH), 19.9 ($CH_2CH\underline{C}H_3$), 12.0 ($\underline{C}H_3CHCHO$).

2.4 Preparation of 13-acetoxyfarnesyl diphosphate (13-OAc FDP, 16)

(6*E*, 10*E*)-2-bromo-2, 6, 10-trimethyl-12-((tetrahydro-2H-pyran-2-yl) oxy) dodeca-6,10-dien-3-ol

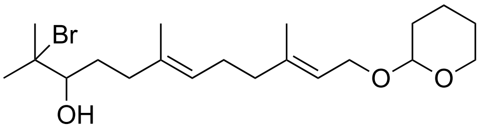

N-Bromosuccinimide (1.9 g, 10.7 mmol) was added in small amounts to a stirred solution of THP-protected farnesol (3.0 g, 9.8 mmol) in a mixture of THF and water (100:40 mL) at 5 °C. Additional water was added dropwise when needed to retain the cloudiness of the solution. Once TLC analysis (8:2, hexane: ethyl acetate) confirmed that there was no starting material left, the reaction was quenched with brine (50 mL). The layers were separated and the aqueous layer was extracted with diethyl ether (5 x 20 mL). Pooled diethyl ether extracts were washed with saturated aqueous sodium bicarbonate solution (20 mL), brine (30 mL), dried over anhydrous magnesium sulfate, filtered under gravity and concentrated under reduced pressure. The crude oil was purified by flash chromatography on silica gel (4:1, hexane: ethyl acetate) to give the title compound as a colourless oil (2.3 g, 67%).
^{1}H-NMR (400 MHz, $CDCl_3$): δ 5.36 (1 H, t, $J_{H,H}$ = 6.5, $C\underline{H}CH_2O$), 5.20 (1 H, t, $J_{H,H}$ = 6.5, $CH_3CC\underline{H}CH_2CH_2$), 4.63 (1 H, t, $J_{H,H}$ = 3.5, $OC\underline{H}O$), 4.24 (1 H, dd, $J_{H,H}$ = 12.0 and 6.5, $CCHC\underline{H_A}O$), 4.03 (1 H, dd, $J_{H,H}$ = 12.0 and 7.5, $CCHC\underline{H_B}O$), 3.97 (1 H, dt, $J_{H,H}$ = 11.5 and 1.5, $OHC\underline{H}CH_2$), 3.92-3.49 (2 H, m, $OC\underline{H_2}CH_2$), 2.35-2.04 (8 H, m, 2 x $CCHC\underline{H_2}C\underline{H_2}$), 1.86-1.51 (6 H, m, $OCHC\underline{H_2}C\underline{H_2}C\underline{H_2}$), 1.68, 1.59, 1.34, 1.33 (4 x 3 H, 4 x s, 2 x $CH_2CHCC\underline{H_3}$ and 2 x $OHCHCC\underline{H_3}$). **^{13}C-NMR** (125 MHz, $CDCl_3$): δ 139.4 and 133.5 (2 x $CH_3\underline{C}CH$), 125.9 and 120.9 (2 x $CH_3C\underline{C}H$), 97.9 ($O\underline{C}HO$), 94.8 ($CH_3C\underline{C}HOH$), 63.9 and 62.5 (2 x $CHO\underline{C}H_2$), 39.7 ($CH_3\underline{C}Br$), 38.3 and 32.2 (2 x $CH_3C\underline{C}H_2$), 30.9 ($OCH\underline{C}H_2$), 26.7 and 26.4 (2 x $CCH\underline{C}H_2CH_2$), 26.0 and 25.7 (2 x $OHCHC\underline{C}H_3$), 25.4 ($OCH_2\underline{C}H_2$), 19.8 ($OCH_2CH_2\underline{C}H_2$), 16.6 and 16.0 (2 x $CH_2C\underline{C}H_3$). **HRMS** (ES^+): calculated for $C_{20}H_{35}O_3Br[Na]^+$: 425.1667, found: 425.1660.

2-(((2*E*, 6*E*)-9-(3, 3-dimethyl oxiran-2-yl)-3, 7-dimethyl nona-2, 6-dien-1-yl) oxy) tetrahydro-2H-pyran

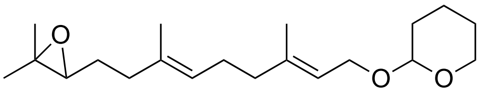

The newly obtained bromohydrin (3.5 g, 8.7 mmol) was added to a solution of potassium carbonate (2.4 g, 17.4 mmol) dissolved in methanol (50 mL). The mixture was stirred for 1 h at room temperature. The methanol was removed and the residual oil was dissolved in diethyl ether (20 mL). A solution of saturated aqueous sodium bicarbonate solution (80 mL) was added to dilute the solution. The layers were separated and the aqueous layer was washed with additional diethyl ether (5 x 20 mL). Combined ether extracts were washed with brine (20 mL), dried over anhydrous magnesium sulfate, filtered under gravity and concentrated under reduced pressure. The crude oil was purified by

flash chromatography on silica gel (4:1, hexane: ethyl acetate) to yield the title epoxide as a colourless oil (2.3 g, 81%).

^{1}H-NMR (300 MHz, $CDCl_3$): δ 5.36 (1 H, t, $J_{H,H}$ = 7.0, $C\underline{H}CH_2O$), 5.15 (1 H, t, $J_{H,H}$ = 6.5, $CH_3CC\underline{H}CH_2CH_2$), 4.62 (1 H, t, $J_{H,H}$ = 3.5, $OC\underline{H}O$), 4.24 (1 H, dd, $J_{H,H}$ = 12.0 and 6.5, $CCHC\underline{H}_AO$), 4.01 (1 H, dd, $J_{H,H}$ = 12.0 and 7.5, $CCHC\underline{H}_BO$), 3.92-3.47 (2 H, m, $OC\underline{H_2}CH_2$), 2.70 (1 H, t, $J_{H,H}$ = 6.0, $COC\underline{H}$), 2.15-1.80 (8 H, m, 2 x $CCHC\underline{H_2}C\underline{H_2}$), 1.72 - 1.49 (6 H, m, $OCHC\underline{H_2}C\underline{H_2}C\underline{H_2}$), 1.67, 1.61, 1.29 and 1.25 (4 x 3 H, 4 x s, 4 x $C\underline{H_3}$). **^{13}C-NMR** (125 MHz, $CDCl_3$): δ 140.3 and 134.5 (2 x $CH_3\underline{C}CH$), 124.7 and 120.8 (2 x $CH_3C\underline{C}H$), 98.0 ($O\underline{C}HO$), 64., 63.8, 62.5 and 58.5 (2 x $O\underline{C}H$ and 2 x $O\underline{C}H_2$), 39.7 and 36.5 (2 x $CH_3C\underline{C}H_2$), 30.9 ($OCH\underline{C}H_2$), 27.6 and 26.5 (2 x $CCH\underline{C}H_2CH_2$), 25.7 ($OCH_2\underline{C}H_2$), 25.1 ($OC\underline{C}H_3$), 19.8 ($OCH_2CH_2\underline{C}H_2$), 18.9 ($OC\underline{C}H_3$), 16.6 and 16.2 (2 x $CH_2C\underline{C}H_3$). **HRMS** (ES^+): calculated for $C_{20}H_{34}O_3[Na]^+$: 345.2406, found: 345.2393.

(4*E*, 8*E*)-4, 8-dimethyl-10-((tetrahydro-2*H*-pyran-2-yl) oxy) deca-4, 8-dienal

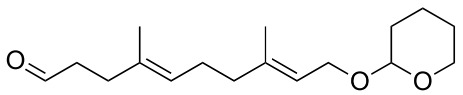

A solution of the above epoxide (330 mg, 1.0 mmol) in a mixture of THF and water (10:1 mL) was treated sequentially at 0 °C with sodium periodate (130 mg, 0.6 mmol) and periodic acid (260 mg, 1.1 mmol). The resulting biphasic solution was stirred at 0 °C for 10 min and then warmed to room temperature. After 1 h, the reaction was quenched with saturated aqueous sodium bicarbonate solution (25 mL) and poured into brine (25 mL). The biphasic layers were separated and the aqueous layer was extracted with diethyl ether (5 x 20 mL). Combined organic layers were washed with brine (20 mL), dried over anhydrous magnesium sulfate, filtered under gravity and concentrated under reduced pressure. The resulting oil was purified by flash chromatography on silica gel (4:1, hexane: ethyl acetate) to give the title compound as a colourless oil (250 mg, 87%).

^{1}H-NMR (300 MHz, $CDCl_3$): δ 9.75 (1 H, t, $J_{H,H}$ = 2, $CH_2C\underline{H}O$), 5.34 (1 H, t, $J_{H,H}$ = 6.5, $C\underline{H}CH_2O$), 5.13 (1 H, t, $J_{H,H}$ = 6.5, $CC\underline{H}CH_2CH_2$), 4.62 (1 H, t, $J_{H,H}$ = 4.0, $OC\underline{H}O$), 4.24 (1 H, dd, $J_{H,H}$ = 12.0 and 6.5, $CCHC\underline{H}_AO$), 4.01 (1 H, dd, $J_{H,H}$ = 12.0 and 7.5, $CCHC\underline{H}_BO$), 3.93-3.47 (2 H, m, $OC\underline{H_2}CH_2$), 2.54-2.48 (2 H, m, $OCHC\underline{H_2}CH_2C$), 2.31 (2 H, t, $J_{H,H}$ = 7.5, $OCHCH_2C\underline{H_2}C$), 2.13-2.03 (4 H, m, $CCHC\underline{H_2}C\underline{H_2}$), 1.75-1.50 (6 H, m, $OCHC\underline{H_2}C\underline{H_2}C\underline{H_2}$), 1.67 and 1.61 (2 x 3 H, 2 x s, 2 x $C\underline{H_3}$). **^{13}C-NMR** (125 MHz, $CDCl_3$): δ 202.9 ($CCH_2CH_2\underline{C}HO$), 140.1 and 133.4 (2 x $CH_3\underline{C}CH$), 125.2 and 121.0 (2 x $CH_3C\underline{C}H$), 98.0 ($O\underline{C}HO$), 63.8 and 62.5 (2 x $CHO\underline{C}H_2$), 42.3 ($CCH_2\underline{C}H_2CHO$), 39.6 and 32.0 (2 x $CH_3C\underline{C}H_2$), 30.9 ($OCH\underline{C}H_2$), 26.3 ($CCH\underline{C}H_2CH_2$), 25.7 ($OCH_2\underline{C}H_2$), 19.8 ($OCH_2CH_2\underline{C}H_2$), 16.6 and 16.3 (2 x $CH_2C\underline{C}H_3$). **HRMS** (ES^+): calculated for $C_{17}H_{28}O_3[Na]^+$: 303.1936, found: 303.1943.

(2*Z*, 6*E*, 10*E*)-ethyl-2, 6, 10-trimethyl-12-((tetrahydro-2*H*-pyran-2-yl) oxy) dodeca-2, 6, 10-trienoate

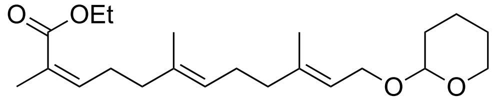

To a stirred solution of the above aldehyde (123 mg, 0.36 mmol) in anhydrous THF (5 mL) was added 18-crown-6 (400 μL, 1.8 mmol) and the solution was cooled to -78 °C. Potassium bis(trimethylsilyl)amide (KHMDS) (1 M in THF, 360 μL, 0.36 mmol) was added dropwise over 5 min and then was left to stir for 15 min. Ethyl trifluoroethyl phosphonopropionate (100 mg, 0.36 mmol) was dissolved in a minimal amount of anhydrous THF (5 mL) and added slowly over 5 min to the reaction. The reaction was stirred for 1 h before being quenched with saturated aqueous ammonium chloride solution (20 mL). The layers were separated and the aqueous layer was extracted with diethyl ether (3 x 20 mL). Combined ether extracts were washed with brine (10 mL), dried over magnesium sulfate, filtered under gravity and concentrated under reduced pressure. The crude product was purified by flash chromatography on silica gel (9:1, hexane: ethyl acetate) to the title compound as a colourless oil (36 mg, 41%).

^{1}H-NMR (300 MHz, $CDCl_3$): δ 5.90 (1 H, t, $J_{H,H}$ = 7.5, $CH_2C\underline{H}CC$), 5.36 (1 H, t, $J_{H,H}$ = 6.5, $C\underline{H}CH_2O$), 5.13 (1 H, t, $J_{H,H}$ = 6.5, $CC\underline{H}CH_2CH_2$), 4.62 (1 H, t, $J_{H,H}$ = 4.0, $OC\underline{H}O$), 4.27-4.16 (3 H, m, $CCHC\underline{H_A}O$ and $COC\underline{H_2}CH_3$), 4.19 (2 H, q, $J_{H,H}$ = 7.0, $COC\underline{H_2}CH_3$), 4.02 (1 H, dd, $J_{H,H}$ = 12.0 and 7.5, $CCHC\underline{H_B}O$), 3.93-3.47 (2 H, m, $OC\underline{H_2}CH_2$), 2.59-2.51 (2 H, m, $CCHC\underline{H_2}CH_2$), 2.16-2.03 (6 H, m, $CCHC\underline{H_2}C\underline{H_2}$ and 2 x $CCHCH_2C\underline{H_2}$), 1.88 and 1.68 (2 x 3 H, 2 x s, 2 x $C\underline{H_3}$), 1.64-1.49 (6 H, m, $OCHC\underline{H_2}C\underline{H_2}C\underline{H_2}$), 1.59 (3 H, s, $C\underline{H_3}$), 1.30 (3 H, t, $J_{H,H}$ = 7.0, $CH_2C\underline{H_3}$). **^{13}C-NMR** (75 MHz, $CDCl_3$): δ 168.4 ($\underline{C}OCH_2CH_3$), 142.8, 140.4 and 134.7 (3 x $CH_3\underline{C}CH$), 127.3, 124.7 and 120.8 (3 x $CH_3C\underline{C}H$), 98.0 ($O\underline{C}HO$), 63.8 and 62.5 (2 x $CHO\underline{C}H_2$), 60.2 ($CO\underline{C}H_2CH_3$), 39.8 and 39.3 (2 x $CH_3C\underline{C}H_2$), 30.9 ($OCH\underline{C}H_2$), 28.1 and 26.5 (2 x $CCH\underline{C}H_2CH_2$), 25.7 ($OCH_2\underline{C}H_2$), 20.8 ($OCC\underline{C}H_3$), 19.8 ($OCH_2CH_2\underline{C}H_2$), 16.6 and 16.1 (2 x $CH_2C\underline{C}H_3$), 14.5 ($COCH_2\underline{C}H_3$). **HRMS** (AP^+): calculated for $C_{22}H_{36}O_4[Na]^+$: 387.2511, found: 387.2514.

(2*Z*, 6*E*, 10*E*)-2, 6, 10-trimethyl-12-((tetrahydro-2*H*-pyran-2-yl) oxy) dodeca-2, 6, 10-trien-1-ol

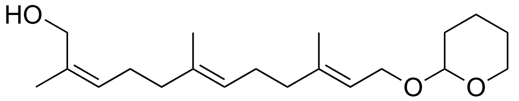

The above conjugated ethyl ester (0.2 g, 0.6 mmol) was cooled to -78 °C and stirred in anhydrous THF (5 mL). Diisobutylaluminium hydride (1 M in toluene, 1.7 mL, 1.7 mmol) was added drop-wise and the reaction was left to stir until TLC analysis (8:2, hexane: ethyl acetate) showed full consumption of starting material. The reaction was quenched with saturated aqueous sodium bicarbonate solution (30 mL) and left to stir for 1 h. The layers were separated and the aqueous layer was washed with diethyl ether (3 x 10 mL), brine (10 mL) and dried over anhydrous magnesium sulfate before being filtered under gravity and concentrated under reduced pressure. The crude oil was purified by flash chromatography on silica gel (4:1, hexane: ethyl acetate) to give the corresponding allylic alcohol as a colourless oil (110 mg, 62%).

^{1}H-NMR (300 MHz, $CDCl_3$): δ 5.36 (1 H, t, $J_{H,H}$ = 7.0, $C\underline{H}CH_2O$), 5.28 (1 H, t, $J_{H,H}$ = 7.0, $OHCH_2CC\underline{H}$), 5.10 (1 H, t, $J_{H,H}$ = 6.5, $CC\underline{H}CH_2CH_2$), 4.63 (1 H, t, $J_{H,H}$ = 4.0, $OC\underline{H}O$), 4.24 (1 H, dd, $J_{H,H}$= 12.0 and 6.5, $CCHC\underline{H_A}O$), 4.11 (2 H, s, $OHC\underline{H_2}$), 4.03 (1 H, dd, $J_{H,H}$= 12.0 and 7.5, $CCHC\underline{H_B}O$), 3.93-3.48 (2 H, m, $OC\underline{H_2}CH_2$), 2.18-1.96 (8 H, m, 2 x $CCHC\underline{H_2}C\underline{H_2}$), 1.79 and 1.68 (2 x 3 H, 2 x s, 2 x $C\underline{H_3}$), 1.61-1.48

(6 H, m, $OCHC\underline{H}_2C\underline{H}_2C\underline{H}_2$), 1.59 (3 H, s, $C\underline{H}_3$). **^{13}C-NMR** (75 MHz, $CDCl_3$): δ 140.3, 135.0 and 134.6 (3 x $CH_3\underline{C}CH$), 128.4, 124.7 and 120.8 (3 x $CH_3C\underline{C}H$), 98.0 ($O\underline{C}HO$), 63.8 and 62.5 (2 x $CHO\underline{C}H_2$), 61.8 ($OH\underline{C}H_2$), 39.9 and 39.7 (2 x $CH_3C\underline{C}H_2$), 30.9 ($OCH\underline{C}H_2$), 26.4 and 25.7 (2 x $CCH\underline{C}H_2CH_2$), 21.5 ($OCH_2\underline{C}H_2$), 19.8 ($OCH_2CH_2\underline{C}H_2$), 16.6, 16.2 and 14.3 (3 x CH_3).

(2*Z*, 6*E*, 10*E*)-2, 6, 10-trimethyl-12-((tetrahydro-2H-pyran-2-yl) oxy) dodeca-2, 6, 10-trien-1-yl acetate

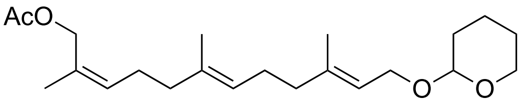

To a stirred solution of the newly obtained allylic alcohol (110 mg, 340 μmol) in dichloromethane (5 mL), was added acetic anhydride (130 μL, 1.4 mmol) and pyridine (110 μL, 1.4 mmol). The reaction was left to stir at room temperature for 24 h and quenched with saturated aqueous sodium bicarbonate solution (10 mL). The biphasic layers were separated and the aqueous layer was washed with diethyl ether (4 x 10 mL). The combined organic layers were washed with brine (5 x 10 mL) and dried over anhydrous magnesium sulfate before being filtered under gravity. The solvent was removed under reduced pressure and the crude oil was purified by flash chromatography on silica gel (8:1, hexane: ethyl acetate) to give the title compound as a colourless oil (119 mg, 96%).

^{1}H NMR (300 MHz, $CDCl_3$): δ 5.40-5.34 (2 H, m, 2 x $C\underline{H}CH_2O$ and $OCH_2CC\underline{H}$), 5.11 (1 H, t, $J_{H,H}$ = 6.5, $CC\underline{H}CH_2CH_2$), 4.62 (1 H, t, $J_{H,H}$ = 4.0, $OC\underline{H}O$), 4.57 (2 H, s, $OC\underline{H}_2CCH$), 4.24 (1 H, dd, $J_{H,H}$ = 12.0 and 6.5, $CCHC\underline{H}_AO$), 4.02 (1 H, dd, $J_{H,H}$ = 12.0 and 7.5, $CCHC\underline{H}_BO$), 3.93-3.47 (2 H, m, $OC\underline{H}_2CH_2$), 2.19-1.96 (8 H, m, 2 x $CCHC\underline{H}_2C\underline{H}_2$), 2.07 (3 H, s, $OCC\underline{H}_3$), 1.75-1.49 (6 H, m, $CHC\underline{H}_2C\underline{H}_2C\underline{H}_2$), 1.74, 1.68 and 1.58 (3 x 3 H, 3 x s, 3 x $CHCC\underline{H}_3$). **^{13}C NMR** (75 MHz, $CDCl_3$): δ 171.4 ($O\underline{C}O$), 140.4, 134.7 and 130.7 (3 x $\underline{C}CHCH_2$), 129.9, 124.6 and 120.7 (3 x $C\underline{C}HCH_2$), 98.0 ($O\underline{C}HO$), 63.8 ($C\underline{C}H_2O$), 63.4 and 62.5 (2 x $CHO\underline{C}H_2$), 39.8 and 30.9 (2 x $CCHCH_2\underline{C}H_2$), 26.5 and 25.7 (2 x $CCH\underline{C}H_2CH_2$), 24.7 ($OCH_2\underline{C}H_2CH_2$), 21.6 ($OCH_2CH_2\underline{C}H_2$), 21.2, 19.8, 16.6, 16.1 (4 x $C\underline{C}H_3$). **HRMS** (ES^+): calculated for $C_{22}H_{36}O_4[Na]^+$: 387.2511, found: 387.2506.

(2*Z*, 6*E*, 10*E*)-12-hydroxy-2, 6, 10-trimethyl dodeca-2, 6, 10-trien-1-yl acetate

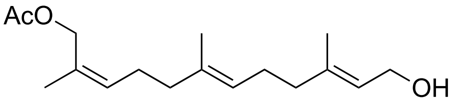

The above allylic acetate (110 mg, 0.3 μmol) and *p*-toluenesulfonic acid (6 mg, 30 μmol) were mixed in methanol (5 mL) and stirred at room temperature for 1 h until TLC analysis (8:2, hexane: ethyl acetate) confirmed complete consumption of starting material. The solvent was removed under reduced pressure and the residual oil was dissolved with diethyl ether (10 mL) and washed with saturated aqueous sodium bicarbonate solution (3 x 10 mL) and brine (10 mL). The resulting solution was dried over anhydrous magnesium sulfate, filtered under gravity and concentrated under reduced pressure to the title compound (13-acetoxyfarnesol) as a colourless oil (84 mg, 99%). The product was judged by ^{1}H NMR spectroscopy to be sufficiently pure to be carried forward without further purification.

¹H-NMR (300 MHz, $CDCl_3$): δ 5.41 (1 H, t, $J_{H,H}$ = 7.0, OCH_2CCH), 5.38 (1 H, t, $J_{H,H}$ = 7.5, CCH), 5.11 (1 H, t, $J_{H,H}$ = 6.5, $CCHCH_2$), 4.57 (2 H, s, OCH_2C), 4.15 (2 H, d, $J_{H,H}$ = 7.0, $CHCH_2O$), 2.18-1.98 (8 H, m, 2 x $CCHCH_2CH_2$), 2.07 (3 H, s, $OCCH_3$), 1.74, 1.68 and 1.59 (3 x 3 H, 3 x s, 3 x CH_2CCH_3). **¹³C-NMR** (75 MHz, $CDCl_3$): δ 171.45 (OCO), 139.8, 134.9 and 130.7 (3 x $CCHCH_2$), 129.9, 124.4 and 123.7 (3 x $CCHCH_2$), 63.4 (CH_2OC), 59.6 (CH_2OH), 39.8 and 39.6 (2 x $CCHCH_2CH_2$), 26.5 and 26.4 (2 x $CCHCH_2CH_2$), 21.6, 21.2, 16.4 and 16.2 (4 x CCH_3). **HRMS** (ES^+): calculated for $C_{17}H_{28}O_3[Na]^+$: 303.1936, found: 303.1930.

(2*Z*, 6*E*, 10*E*)-12-bromo-2, 6, 10-trimethyl dodeca-2, 6, 10-trien-1-yl acetate

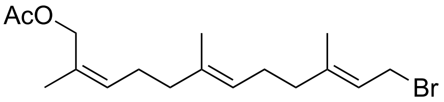

To a stirred solution of 13-acetoxyfarnesol (90 mg, 0.3 mmol) in anhydrous THF (5 mL) at -10 °C, was added phosphorous tribromide (15 μL, 0.2 mmol), and the reaction was left to stir for 1 h. When TLC analysis (8:2, hexane: ethyl acetate) confirmed complete consumption of starting material, the phosphorous tribromide and THF were removed under reduced pressure. The residual product was diluted in diethyl ether (10 mL) and washed with saturated aqueous ammonium bicarbonate solution (3 x 20 mL) and brine (10 mL). The resulting solution was dried over anhydrous magnesium sulfate, filtered under gravity and concentrated under reduced pressure to give the title compound as a colourless oil (40 mg, 36%).

¹H-NMR (300 MHz, $CDCl_3$): δ 5.52 (1 H, t, $J_{H,H}$ = 8.5, CCH), 5.37 (1 H, t, $J_{H,H}$ = 7.0, CCH), 5.08 (1 H, t, $J_{H,H}$ = 8.0, $CCHCH_2$), 4.57 (2 H, s, OCH_2C), 4.02 (2 H, d, $J_{H,H}$ = 8.5, $CHCH_2Br$), 2.19-1.96 (8 H, m, 2 x $CCHCH_2CH_2$), 2.07 (3 H, s, $OCCH_3$), 1.74, 1.72 and 1.58 (3 x 3 H, 3 x s, 3 x $CHCCH_3$).

Tris-(tetrabutyl ammonium) 13-acetoxyfarnesyl diphosphate (16)

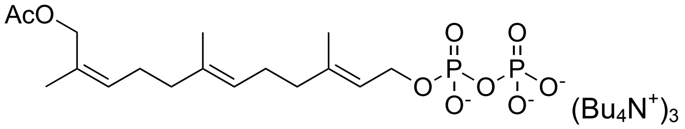

A solution of the above bromide (40 mg, 0.1 mmol) in anhydrous acetonitrile (5 mL) was treated with $(Bu_4N)_3HP_2O_7$ (210 mg, 0.2 mmol). The reaction was left for 48 h to stir under nitrogen. The solvent was removed under reduced pressure and the resulting crude oil was purified by flash chromatography on silica gel (6:2.5:0.5, isopropanol: water: ammonium hydroxide). Fractions that contained the diphosphate and therefore gave a spot with TLC (6:3:1, isopropanol: water: ammonium hydroxide) were collected. The isopropanol was removed under reduced pressure and the remaining solution was lyophilized to give **16** as colourless oil (30 mg, 20%).

¹H-NMR (500 MHz, D_2O): δ 5.49 (1 H, t, $J_{H,H}$ = 7.5, CCH), 5.45 (1 H, t, $J_{H,H}$ = 7.0, CCH), 5.20 (1 H, t, $J_{H,H}$ = 6.5, $CCHCH_2$), 4.62 (2 H, s, OCH_2C), 4.48 (2 H, t, $J_{H,H}$ = 7.0, $CHCH_2O$), 3.21-3.17 (24 H, m, 12 x $NCH_2CH_2CH_2CH_3$), 2.22-2.03 (11 H, m, 2 x $CCHCH_2CH_2$ and $OCCH_3$), 2.03 (3 H, s, $OCCH_3$), 1.73-1.61 (33 H, m, 12 x $NCH_2CH_2CH_2CH_3$ and 3 x CH_2CCH_3), 1.39-1.32 (24 H, m, 12 x

$NCH_2CH_2C\underline{H_2}CH_3$), 0.94 (36 H, t, $J_{H,H}$ = 7.0, 12 x $NCH_2CH_2CH_2C\underline{H_3}$). **^{31}P-NMR** (121 MHz, D_2O): δ -10.21 (m), -10.73 (m). **HRMS** (ES^-): calculated for $C_{17}H_{27}O_9P_2[2H]^-$: 439.1287, found: 439.1286.

2.5 Synthesis of dihydroartemisinic methyl ester (18) from dihydroartemisinic aldehyde (4).

Dihydroartemisinic acid (6)

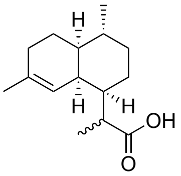

Dihydroartemisinic aldehyde (9 mg, 0.04 mmol) was dissolved in a mixture of *t*BuOH (0.4 mL) and 2-methyl-2-butene (0.05 mL) and cooled down to 0 ºC. A solution of $NaClO_2$ (22 mg, 0.24 mmol) and NaH_2PO_4 (48 mg, 0.40 mmol) in deionised water (0.25 mL) was added and the mixture was stirred at 0 ºC for 45 min. The reaction mixtrure was diluted with diethyl ether and extracted with additional diethyl ether (3 x 5 mL). The combined organic phases were washed with brine, dried over Na_2SO_4 and concentrated under reduced pressure. The crude was purified by flash column chromatography on silica (diethyl ether/petroleum ether: 1:4 to 1:1) to give dihydroartemisinic acid (**6**) as colourless oil (9 mg, 93% yield).

(11*S*-6) **^{1}H-NMR** (300 MHz, $CDCl_3$): δ (ppm) 5.29 (1 H, bs, $CH_3C{=}C\underline{H}$), 2.58-2.50 (1 H, m, 1 x $C{=}CHC\underline{H}$), 2.41 (1 H, bs, 1 x $OCC\underline{H}$),1.91-1.78 (3 H, m, 3 x $CH_3CC\underline{H_2}C\underline{H_b}$), 1.75-1.54 (3 H, m, 3 x $CH_3CCH_2C\underline{H_a}$ & $OCCHC\underline{H}$ & $CH_3CHC\underline{H_b}$), 1.42 (1 H, bs, 1 x $CH_3C\underline{H}CH_2$), 1.25-1.17 (2 H, m, 2 x $OCCHCHC\underline{H_a}$ & $CH{=}CCH_2CH_2C\underline{H}$), 1.00-0.92 (2 H, m, 2 x $OCCHCHC\underline{H_b}$ & $CH_3CHC\underline{H_a}$), 1.63 (3 H, s, CH_3), 1.23 (3 H, d, *J* = 8.0, CH_3), 0.87 (3 H, d, *J* = 6.5, CH_3). **^{13}C-NMR** (500 MHz, $CDCl_3$): δ (ppm) 182.2, 135.5,120.6, 45.0, 41.8, 39.4, 35.6, 29.9, 27.8, 26.7, 25.7, 24.9, 24.1, 19.9, 16.4.

(11*R*-6) **^{1}H-NMR** (500 MHz, $CDCl_3$): δ (ppm) 5.12 (1 H, bs, $C\underline{H}$), 2.58-2.50 (2 H, m, 2 x $C{=}CHC\underline{H}$ & $OCC\underline{H}$), 1.91-1.78 (3 H, m, 3 x $CH_3CC\underline{H_2}C\underline{H_b}$), 1.75-1.54 (3 H, m, 3 x $CH_3CCH_2C\underline{H_a}$ & $OCCHC\underline{H}$ & $CH_3CHC\underline{H_b}$), 1.42 (1 H, bs, 1 x $CH_3C\underline{H}CH_2$), 1.25-1.17 (2 H, m, 2 x $OCCHCHC\underline{H_a}$ & $CH{=}CCH_2CH_2C\underline{H}$), 1.00-0.92 (2 H, m, 2 x $OCCHCHC\underline{H_b}$ & $CH_3CHC\underline{H_a}$), 1.63 (3 H, s, CH_3), 1.21 (3 H, d, *J* = 7.0, CH_3), 0.87 (3 H, d, *J* = 6.5, CH_3). **^{13}C-NMR** (500 MHz, $CDCl_3$): δ (ppm) 183.7, 136.2, 119.5, 43.8, 41.9, 41.6, 36.6, 35.4, 27.9, 27.6, 26.8, 25.9, 24.0, 19.9, 15.3.

Dihydroartemisinic methyl ester (18)

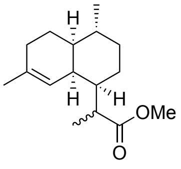

Acid **6** (6 mg, 0.025 mmol) was dissolved in a toluene (0.30 mL) and methanol (0.20 mL) mixture. $TMSCH_2N_2$ (0.025 mL, 2.0 M in diethyl ether) was added and the micture was left to stir at room temperature for 30 min. The mixture was diluted with diethyl ether, quenched with acetic acid (10%) and extracted with additional diethyl ether (3 x 5 mL). Combined organic phases were washed with

brine, dried over Na_2SO_4 and concentrated under reduced pressure. The crude was purified by flash column chromatography on silica (diethyl ether/petroleum ether: 1:9 to 1:4) to give compound **18** as colourless oil (6 mg, 94% yield).

(11*S*-**18**) ^{1}**H-NMR** (500 MHz, CDCl3): δ 5.27 (1 H, bs, $CH_3C{=}C\underline{H}$), 3.73 (3 H, s, CH_3OC), 2.54-2.49 (1 H, m, $OCC\underline{H}CHCH$), 2.24 (1 H, br, $OCCHCHC\underline{H}$), 1.89-1.74 (3 H, m, $CH_3CCH_2C\underline{H}_b$), 1.62 (3 H, s, $CH{=}CCH_3$), 1.66-1.55 (3 H, m, $CH_3CCH_2C\underline{H}_a$ and $OCCHCH$ and $CH_3CHC\underline{H}_b$), 1.51-1.43 (3 H, m, CH_3CHCH_2, $OCCHCHC\underline{H}_a$ and $CH{=}CCH_2CH_2C\underline{H}$), 1.16 (3 H, br, $CHC\underline{H}_3$), 0.95-0.93 (2 H, m, $CH_3CHC\underline{H}_aC\underline{H}_b$), 0.86 (3 H, br, $CHC\underline{H}_3$). ^{13}**C-NMR** (125 MHz, $CDCl_3$): δ 177.4, 135.0, 120.4, 51.4, 45.1, 41.6, 39.2, 35.4, 30.3, 27.5, 26.5, 25.5, 24.8, 23.8, 19.7, 16.2.

(11*R*-**18**) ^{1}**H-NMR** (500 MHz, CDCl3): δ 5.12 (1 H, bs, $CH_3C{=}CH$), 3.73 (3 H, s, CH_3OC), 2.54-2.49 (2 H, m, $OCCHCHCH$), 1.89-1.74 (3 H, m, $CH_3CCH_2C\underline{H}_b$), 1.62 (3 H, s, $CH{=}CCH_3$), 1.66-1.55 (3 H, m, $CH_3CCH_2C\underline{H}_a$ and $OCCHC\underline{H}$ and $CH_3CHC\underline{H}_b$), 1.51-1.43 (3 H, m, $CH_3C\underline{H}CH_2$, $OCCHCHC\underline{H}_a$ and $CH{=}CCH_2CH_2C\underline{H}$), 1.16 (3 H, br, $CHC\underline{H}_3$), 0.95-0.93 (2 H, m, $CH_3CHC\underline{H}_aC\underline{H}_b$), 0.86 (3 H, br, $CHC\underline{H}_3$). ^{13}**C-NMR** (125 MHz, $CDCl_3$): δ 178.2, 125.5, 119.4, 51.4, 43.9, 42.1, 41.7, 36.4, 35.2, 27.6, 27.4, 26.6, 25.7, 23.8, 19.7, 15.1.

3. Preparation of amorphadiene synthase (ADS)

Cloning of ADS gene into pET21d plasmid. The gene encoding for amorphadiene synthase (ADS) from *Artemisia annua* was obtained from the gene bank (JF951730). The gene was supplied in a pTrc99a vector between the NcoI and BamHI restriction sites (pTrc-ADS). pET21d vector and pTrc-ADS were digested with *Nco*I and *BamH*I restriction endonucleases (0.1 μL of each enzyme, 1 μL of buffer, 10 μL of plasmid, 1 h, 37 °C), and the fragments corresponding to an open pET21d and ADS gene were ligated using T4 DNA ligase following the manufacturer's protocol (1:2 molar ratio of pET: ADS, 0.1 μL enzyme, 2 μL of buffer, H_2O to make total volume to 20 μL) to give a new plasmid pET21d-ADS. Supercompetent *E. coli* XL1-blue cells were transformed with 5 μL of ligated DNA and stored on ice (30 minutes) before being heat shocked in a water bath (40 °C, 40 s) and placed back into the ice for another 2 min. LB medium (1 mL) was added and the solution was shaken for 60 min (37 °C, 150 rpm). The cells were harvested by centrifugation (3400 *g*, 1 minute) and spread on an agar plate containing ampicillin (100 μg/mL) after resuspending in a minimum amount of buffer. Plates were incubated overnight at 37 °C and then stored at 4 °C. A single colony from the agar plate was used to inoculate 15 mL of LB medium containing ampicillin (100 μg/mL). The culture was incubated overnight (37 °C) and the following day centrifuged (3220 *g*, 8 min). The pellet was purified using a QIAprep Spin Miniprep Kit (QIAprep Miniprep Handbook-2005). The resulting 50 μL DNA solution was stored at -20 °C. Ligation of the ADS gene into the pET21d vector was confirmed by DNA sequence analysis from Eurofin.

Addition of a C-terminal 6xHis tag to ADS. A single nucleotide deletion was required to bring the 6xHis coding sequence of pET21d in frame with the ADS coding sequence. A Quickchange site-

directed mutagenesis kit was used to introduce the desired deletion according to the manufacturer's instructions. The primers used for the deletion were as follows: 5'-CGATGTCCATCTGTCCCGGGGATCC -3' and 5'-GCTACAGGTAGACAGGGCCCCTAGG -3'. Polymerase chain reaction (PCR) (4 h) was carried out. Samples were then digested with *Dpn*I (1 h, 37 °C).

Plasmids were transformed into *E. coli* XL1-blue cells. A colony from the plate was selected and used to inoculate 15 mL of LB medium containing ampicillin (100 μg/mL). The resulting overnight cultures were centrifuged (3220 *g*, 8 min) and the resulting pellets were purified using *QIAprep Spin Miniprep kit* (QIAprep Miniprep Handbook-2005). The resulting 50 μL DNA solutions were stored at -20 °C. DNA was sequenced to confirm that the site-directed mutagenesis had been successful.

Transformation of competent *E. coli* cells. Plasmid pET21dADS was transformed into competent *E. coli* BL21-(DE3)-Codon Plus RP (EcBL21RP). The plasmid solution (1 μl) was added to the cell suspension (50 μl) and was stored on ice (30 min) before being heat shocked in a water bath (42 °C, 40 s) and placed back into the ice for another 2 min. LB medium (1 mL) was added and the solution was shaken for 60 min (37 °C, 150 rpm). The cells were then harvested by centrifugation (3300 *g*, 1 minute) and spread on an agar plate containing ampicillin (100 μg/mL) after resuspending in a minimum amount of buffer. Plates were incubated overnight at 37 °C and then stored at 4 °C.

Expression of ADS gene. A single colony from the agar plate of *Ec*BL21RP cells was used to inoculate LB medium (100 mL) containing ampicillin (100 μg/ml). The culture was shaken overnight (37 °C, 150 rpm). The resulting cell suspension was used to inoculate LB medium (6 x 500 mL, 10 mL of cell suspension in each flask) containing ampicillin (100 μg/mL). The cells were grown (37 °C, 150 rpm) until the OD_{600} reached 0.5. Isopropyl β-D-1-thiogalactopyranoside (IPTG) (0.048 g, 0.4 mM) was added to each culture and shaking continued (20 °C, 150 rpm) for an additional 6 hours. The cells were harvested by centrifugation (4500 *g*, 10 minutes) and the resulting pellets were stored at -20 °C.

Purification of ADS. Cell pellets were re-suspended in 50 mL of cell lysis buffer (50 mM Tris-Base, 500 mM NaCl, 5 mM imidazole, 20 mM βME, 10% (v/v) glycerol, pH 8). Lysozyme (0.5 mg/ mL) was added and the mixture was left to stir for an hour at 5 °C. Cells were disrupted by sonication at 5 °C (38 % amplitude for 3 min with 5 s on/10 s off cycles) and the resulting suspension was centrifuged at 5 °C (17000 *g*, 30 min) Once SDS-polyacrylamide gel electrophoresis confirmed that the majority of the protein was found in the solution, the pellet was discarded and the supernatant solution was loaded onto a Ni Sepharose™ 6 Fast Flow column (12 mL, the column was eluted under gravity controlled drip flow). After 15 minutes, the flow through was eluted and the column was washed with 10 CV of cell lysis buffer followed by a gradient from 5 to 300 mM imidazole (40 CV). Fractions were analyzed by SDS-PAGE. The protein eluted at 100 mM imidazole. The fractions corresponding to a molecular weight of 56000 were pooled, dialyzed overnight against 25 mM HEPES, 100 mM NaCl, 1 mM DTT, pH 7.5 (MWCO 30000) and then concentrated to a final volume of ~ 10 mL (AMICON

system, YM 30). Solutions were stored at 4 °C. The concentration of protein was estimated using the method of Bradford.[4,5]

4. Steady state kinetics

Optimisation

The procedure for optimizing incubation time involved the incubation of 6 samples of [1-^{3}H]-FDP (10 μM, specific activity 3.11 mCi/mmol) with ADS (100 nM) in incubation buffer (20 mM HEPES, 5 mM β-ME, 5 mM $MgCl_2$, pH 7.5). The reaction mixtures containing buffer, FDP and ADS were prepared on ice in a total volume of 250 μL and were overlaid with hexane (1 mL). Assays were initiated by the addition of enzyme (2 μL, 12.5 μM stock solution) to the assay mixture. The assay mixtures were incubated at 30 °C for varying times; 5, 10, 15, 20, 25, and 30 min. Each reaction was quenched with EDTA (200 μL, 100 mM), briefly vortexed and ice-cooled. The hexane overlay and 2 additional hexane extracts (2 x 750 μL) were passed through a short silica gel column in a Pasteur pipette (~1 g). The column was then eluted with additional hexane (2 x 750 μL). The pooled hexane extracts/eluents were emulsified with Ecoscint^{TM} O fluid (15 mL) and radioactivity was detected using a scintillation counter (Packard 2500 TR^{TM}) in ^{3}H mode for 4 min per sample.

After verifying that 10 min assays were optimal, in turn, enzyme concentration, magnesium ion concentration and pH values were all varied in order to maximize product concentration. Assays were carried out with 50, 100, 150, 200, 300 and 500 nM enzyme, 0, 2, 5, 10, 15 and 20 mM $MgCl_2$ and at pH values of 6.0, 6.5, 7.0, 7.5, 8.0, 8.5 and 9.0. Measurements were made in triplicate. The enzyme and Mg^{2+} concentrations that gave optimal rates were 100 nM and 5 mM, respectively, and the optimal pH was 7.5.

Michaelis-Menten kinetics

Steady state kinetic parameters for ADS were measured using the optimized assay conditions described above and variation of [1-^{3}H]-FDP concentration by fitting of the data to the Michaelis-Menten equation by nonlinear least squares regression in conjunction with the graphical procedures developed by Lineweaver-Burk using the commercial SigmaPlot package.[6-8] Assays (final volume 250 μL) were initiated with the addition of ADS (12.5 μM, 2 μL) to [1-^{3}H]-FDP (240000 dpm/nmol) with concentrations of FDP ranging from 0.2 to 12 μM. The buffer and protocol used was the same as what was used for the optimization tests above.

k_{cat} and K_M values, calculated as an average weighted mean from three separate experiments, were determined to be 0.016 ± 0.006 s^{-1} and 2.25 μM ± 0.30 mM respectively (Figure S1). The catalytic efficiency was calculated at 7.1 x 10^{-3} μM^{-1} s^{-1}. The measured K_M is consistent with that published by Picaud *et al.*,[6] however the k_{cat} was found to be 3-fold faster.

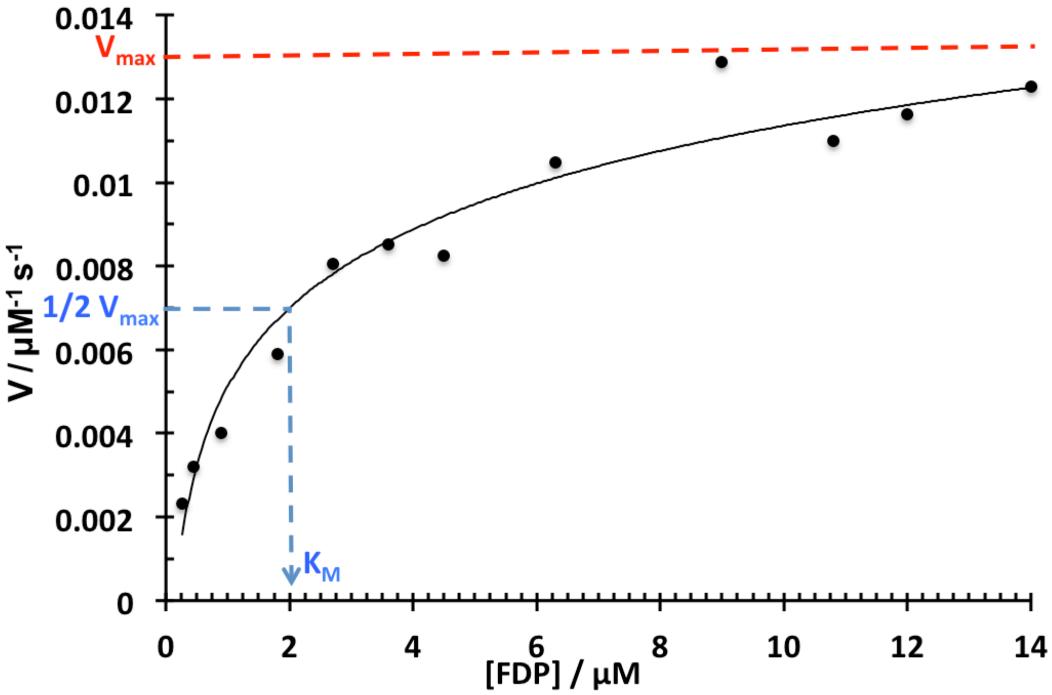

Figure S1. Representative Michaelis-Menten graph of steady-state kinetic parameters of ADS.

5. Optimized synthesis of 12-OH FDP (7). Optimization tables S1 and S2

Table S1. Optimisation of conditions for preparation of 12-hydroxyfarnesyl chloride (**11**).

Entry	Molar equivalents			Yield (%)
	SeO_2	H^+	^tBuOOH	
1	0.1	0.1	5	47
2	0.2	0.2	5	53
3	0.3	0.3	5	62
4	0.5	0.5	5	57
5	1	1	5	8

Table S2. Optimisation of conditions for preparation of 12-hydroxyfarnesyl diphosphate (**7**).

Entry	Molar equivalents of TBDP	Time (Hours)	Yield (%)
1	1.5	24	34
2	2	24	47
3	2	48	51

6. Optimisation on the catalytic turnover of 12-OH FDP (7) to dihydroartemisinic aldehyde (4)

To obtain the optimal conditions for the catalytic turnover, analytical incubations of **7** with ADS at different substrate concentrations and with different organic solvents overlaid for product extraction were carried out (Table S3). The conversions of **7** to **4** were measured using gas chromatography with flame ionisation detection (GC-FID) to analyse the solvent overlays. All incubations with ADS and the novel analogue, **7**, were carried out in parallel to identical incubations of ADS with the natural substrate, FDP (**2**), to compare the reaction efficiency.

All conversions were measured using calibration curves with an analytical standard. Farnesal was used as the standard for measuring the conversion of **7** to **4**. FID response from concentrations of farnesal in the range 10 μM to 4 mM was recorded. Farnesal, which was synthesised from commercially available farnesol, was chosen as it has the same molecular weight and chemical formula as **4**, hence likely to give the same response in the flame ionization detector. The conversions of **7** to **4** were measured using the equation extrapolated from the calibration curve (Figure S1). For the analytical incubations, in addition to a calibration curve, farnesal was also used as an internal standard. The results using both internal and external standards were complementary, with a maximum discrepancy of ca. 4 %. Similarily, to measure the conversions of the natural substrate FDP to amorphadiene, commercially available α-humulene was used as a standard, with concentrations also ranging from 10 μM to 4 mM (Figure S1). This was also used as an internal and external standard for the analytical incubations, also providing consistent results.

Table S3. Analytical incubations of 12-OH-FDP (**7**) with ADS.

Incubation	12-OH-FDP (uM)	Solvent overlay	Conversion from first extraction (%)		Second extraction	
			Using calibration curve	Using internal standard	Solvent	Conversion
1	100	Pentane	65.4	67.0	Pentane	4.3
2	200	Pentane	63.7	63.6	Pentane	1.9
3	300	Pentane	65.1	67.6	-	-
4	400	Pentane	62.0	59.3	-	-
5	200	Pentane	62.8	66.5	EtOAc	2.8
6	200	Pentane	71.9	73.3	DCM	4.6
7	200	EtOAc	15.1	17.9	-	-
8	200	DCM	10.1	10.3	-	-
9	200	No overlay	-		EtOAc	19.6
10	200	No overlay	-		DCM	21.6

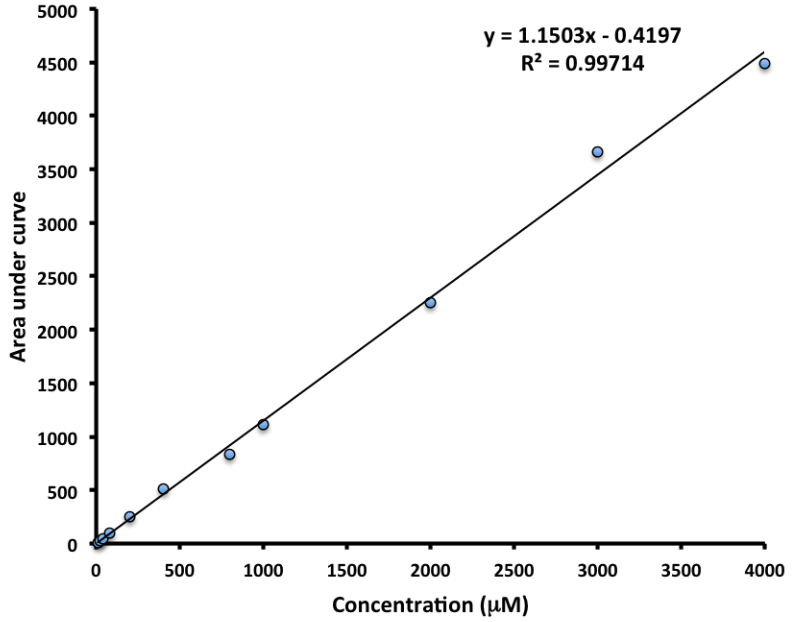

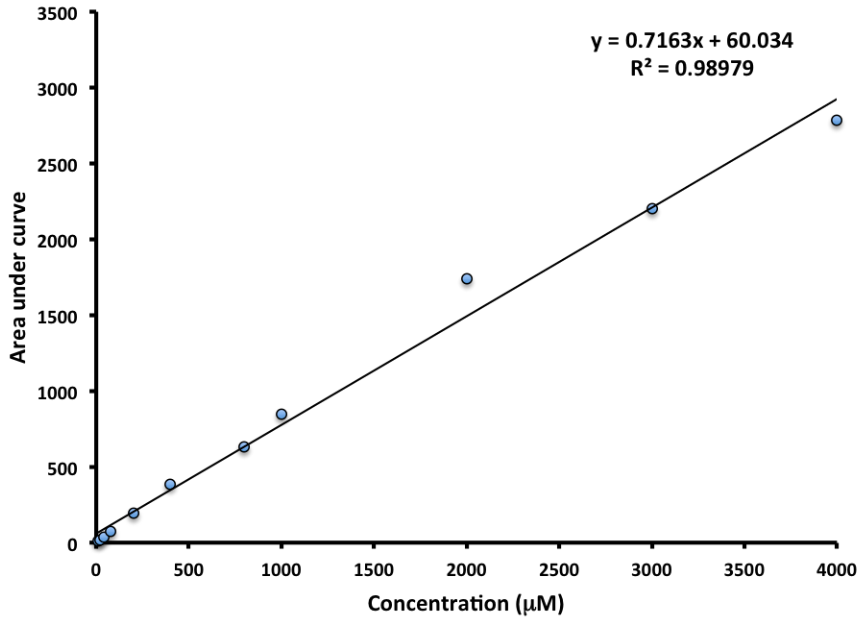

Figure S2. Calibration curve for flame ionization detection response of α-humulene (top) and farnesal (bottom).

7. Enzymatic Incubations

General procedure for analytical incubation of FDP (2) and 12 OH FDP (7) with purified ADS. ADS (1 μM) and substrate (0.4 mM) were both incubated in 250 μL of incubation buffer (20 mM HEPES, 5 mM β-ME, 5 mM $MgCl_2$, pH 7.5), which was overlaid with 1 mL of pentane and left to gently shake overnight at room temperature. The organic layer was separated and analyzed by GC-MS.

General procedure for analytical incubation of FDP (2) and 12 OH FDP (7) with purified ADS in deuterated conditions.

ADS (1 μM) and substrate (0.4 mM) were both lyophilized and resuspended in minimal deuterium oxide before incubating in 250 μL of incubation buffer (20 mM HEPES, 5 mM β-ME, 5 mM $MgCl_2$, pH 7.5). The incubation buffer was prepared in the same manner as above but with deuterium oxide instead of deionized water. The pH was adjusted with the use of NaOD and DCl. Incubations were overlaid with 1 mL of pentane and left to gently shake overnight at room temperature. The organic layer was separated and analyzed by GC-MS.

Procedure for excluding the presence of an ester hydrolase.

12-Acetoxy-FPP (**13**) (12 mM) was incubated for 18 hours with ADS (2 μM) in D_2O without Mg^{2+} and no change in the ^{1}H-NMR of **13** was observed.

Preparative scale incubation using 50 mg of substrate.

To minimize losses of volatile sesquiterpenoid products during incubation and workup, reactions were performed in bottles capped with a rubber seal. Incubations contained incubation buffer (200 mL), substrate (0.4 mM) and enzyme (1 μM). The incubation was then overlaid with 10 mL of pentane. The incubation solution was gently agitated for 24 h at room temperature. An aliquot of the pentane was then removed and analysed by GC-FID for characterisation and a conversion rate.

Preparative scale incubation using 200 mg of substrate.

Incubations contained incubation buffer (800 mL), substrate (0.4 mM) and enzyme (1 μM). The incubation was then overlaid with 20 mL of pentane. The incubation solution was gently agitated for 24 h at room temperature. The following day, an aliquot of the pentane was removed and analysed by GC-FID. The pH of the aqueous buffer was adjusted to 9.5 by addition of 5M sodium hydroxide and the bottle was resealed to allow further stirring for an additional 24 h. The following day, another aliquot of the pentane was removed and analysed by GC-FID. The pentane layer was then separated and the aqueous layer was washed with an additional extraction of pentane (20 mL). The pooled pentane extracts were concentrated under reduced pressure and deuterated chloroform (600 μL) was added for analysis by ^{1}H NMR spectroscopy and GC-MS.

Dihydroartemisinic aldehyde 4 (*R* and *S*)

^{1}H-NMR (500 MHz, $CDCl_3$): δ (ppm) 9.62 (1H, d, $J_{H,H}$ = 3.5, CHO, (11*S*)), 9.57 (1H, d, $J_{H,H}$ = 3.5, CHO, (11*R*)), 5.26 (1H, bs, CH, (11*S*)), 5.12 (1H, bs, CH, (11*R*)), 2.48 (2H, m, 2 x CH, (11*S*) and (11*R*)), 2.39 (2H, m, 2 x CH, (11*S*) and (11*R*)), 1.91-1.25 (20H, m, 2 x $CH_2CH_2CHCHCH_2CH_2$, (11*S*) and (11*R*)), 1.63 (6H, s, 2 x CH_3), 1.08 (3H, d, $J_{H,H}$ = 7.0, CH_3, (11*S*)), 1.06 (3H, d, $J_{H,H}$ = 7.0, CH_3, (11*R*)), 0.87 (6H, d, *J* = 6.5, 2 x CH_3 (11*S*) and (11*R*)).

Amorphadiene 3

^{1}H-NMR (500 MHz, $CDCl_3$): δ (ppm) 5.05 (1H, bs, CH), 4.87 (1H, bs, CH_a), 4.64 (1H, bs, CH_b), 2.55 (1H, m, CH), 1.96-1.37 (11H, m, $CH_2CH_2CHCHCH_2CH_2CH$), 1.74 (3H, s, CH_3), 1.60 (3H, s, CH_3), 0.88 (3H, d, $J_{H,H}$ = 6.5, CH_3).

8. GC-MS

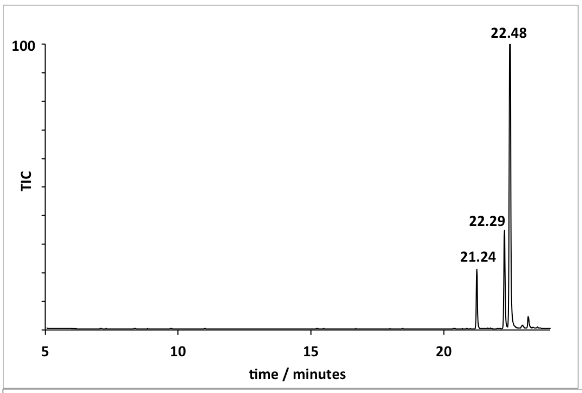

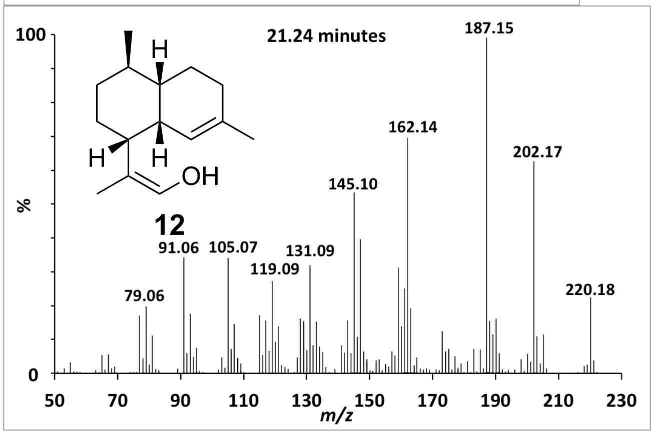

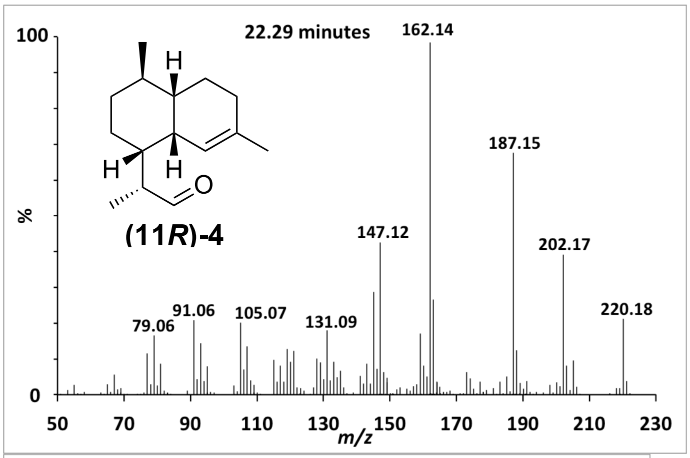

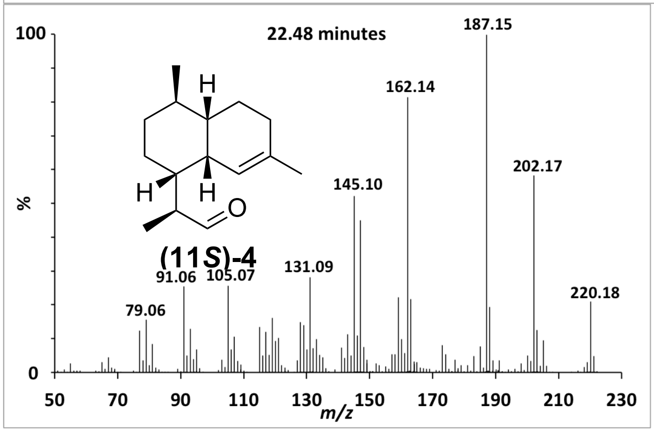

Figure S3. Gas chromatogram (top) and corresponding mass spectra of the organic soluble products isolated from an incubation of ADS with 12-hydroxyfarnesyl diphosphate (12-OH FDP, **7**).

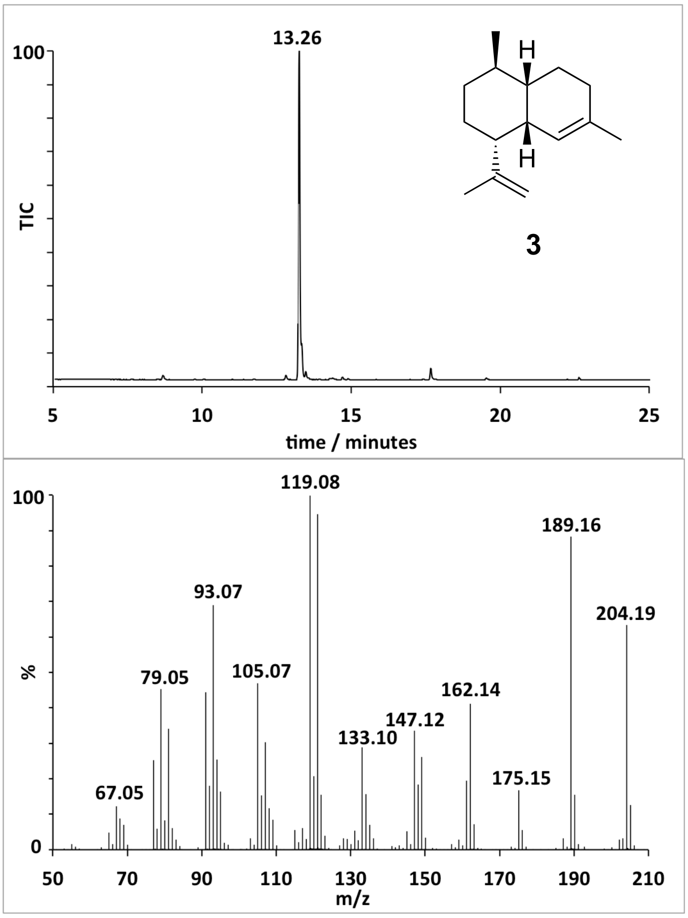

Figure S4. Gas chromatogram (top) and mass spectrum (bottom) of the organic soluble product isolated from an incubation of ADS with farnesyl diphosphate (FDP, **2**).

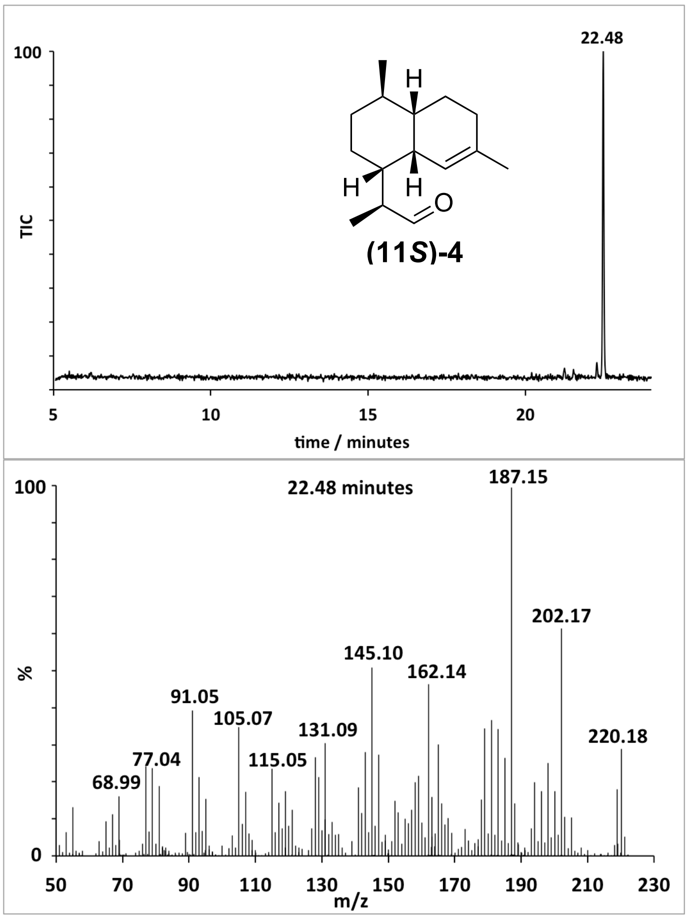

Figure S5. Gas chromatogram (top) and mass spectrum (bottom) of the organic soluble products isolated from an incubation of ADS with 13-acetoxyfarnesyl diphosphate (13-OAc FDP, **16**).

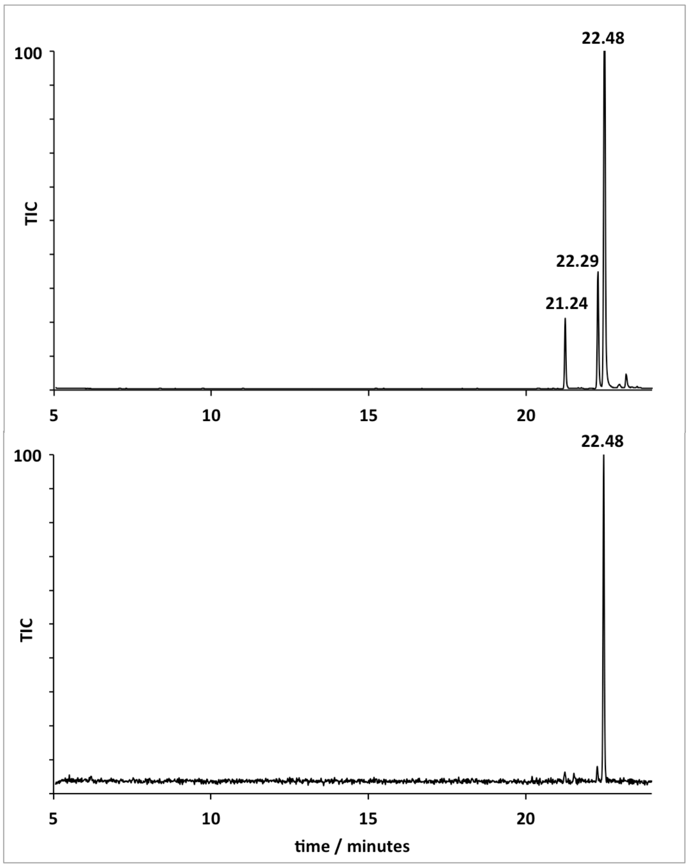

Figure S6. Overlaid gas chromatogram of the organic soluble products isolated from an incubation of ADS with 12-hydroxyfarnesyl diphosphate (12-OH FDP, **7**) 13-acetoxyfarnesyl diphosphate (13-OAc FDP, **16**).

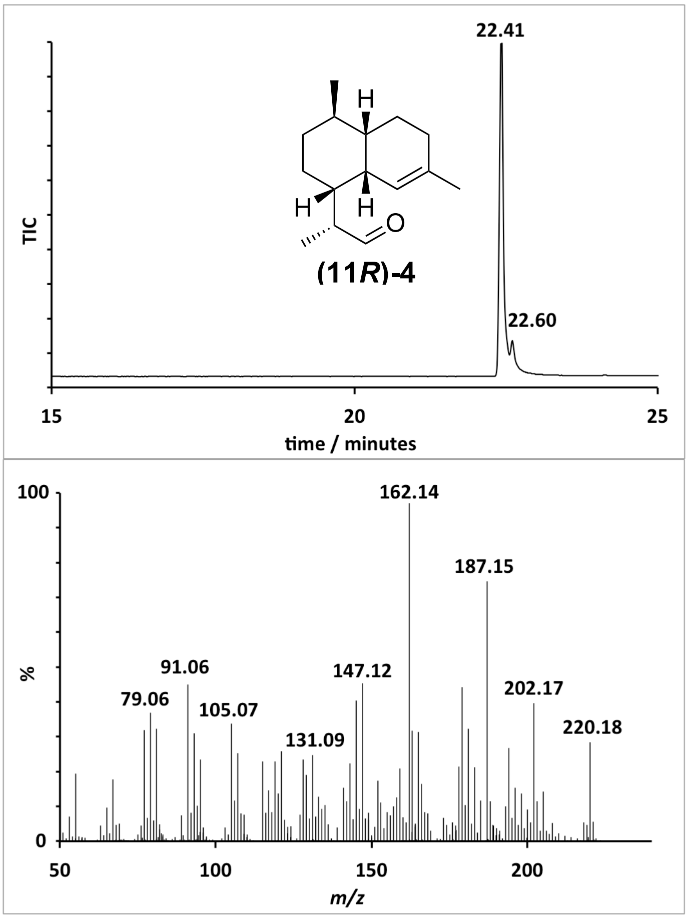

Figure S7. Gas chromatogram (top) and mass spectrum (bottom) of authentic (11*R*)-dihydroartemisinic aldehyde (11*R*-**4**).

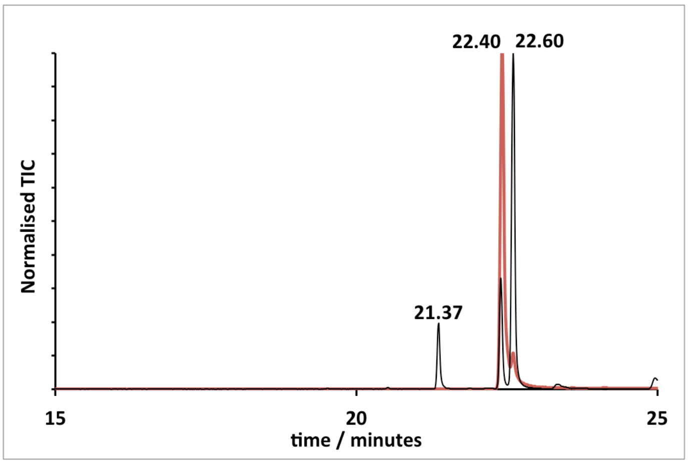

Figure S8. Overlaid gas chromatogram of the organic soluble products isolated from an incubation of ADS with 12-hydroxyfarnesyl diphosphate **7** (black) and an authentic sample of (11*R*)-dihydroartemisinic aldehyde (red)**.**

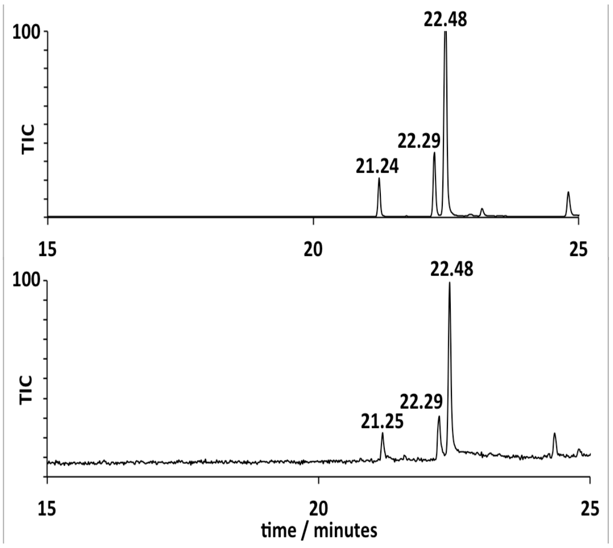

Figure S9. Overlaid gas chromatogram of the organic soluble products isolated from an incubation of ADS with 12-hydroxyfarnesyl diphosphate (**7**, top) and 12-acetoxyfarnesyl diphosphate (**13**, bottom).

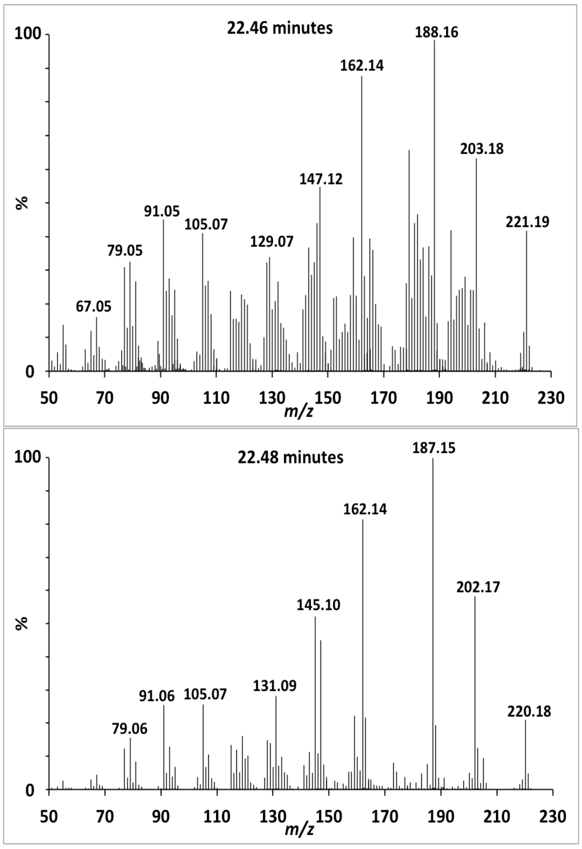

Figure S10. Mass spectra of (11*S*)-[11-$^2H_1$]-**4** (top) and (11*S*)-**4** (bottom). A molecular increase of 1.01 amu can be observed prior to the fragmentation peak (*m/z* = 162.14) in the top spectrum. The MS fragmentation pattern and the diagnostic peaks *m/z* = 221 (M^+), 203 (M^+-H_2O), 188 (M^+-H_2O-CH_3), 162 (M^+-$CH_3CD{=}CHOH$) corresponding to the loss of the oxygenated *d*-isopropyl chain suggested the incorporation of the deuterium label at the C11 position of **4**.

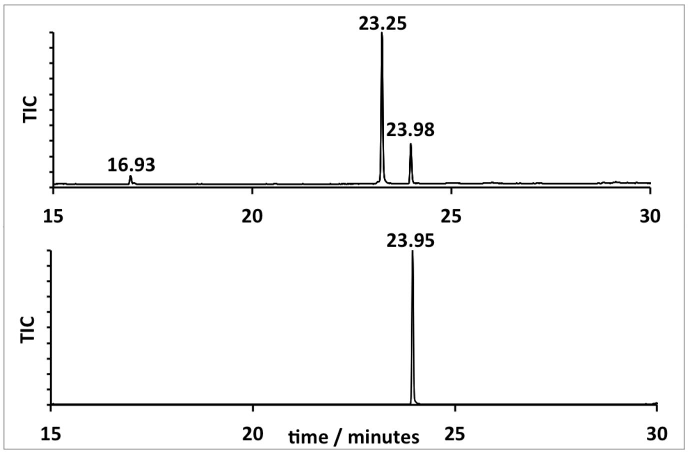

Figure S11. Overlaid gas chromatograms of (11*R*)- and (11*S*)-dihydroartemisinic methyl ester **18** (t_R = 23.98 and 23.25 min, respectively) (top) and an authentic sample of (11*R*)-methyl ester (bottom)**.** The compound with an elution time of 16.93 was identified as a stabiliser in the solvent used.

9. NMR SPECTRA

Figure S12. ^{1}H‑NMR ($CDCl_3$, 500 MHz) of farnesyl diphosphate (**2**).

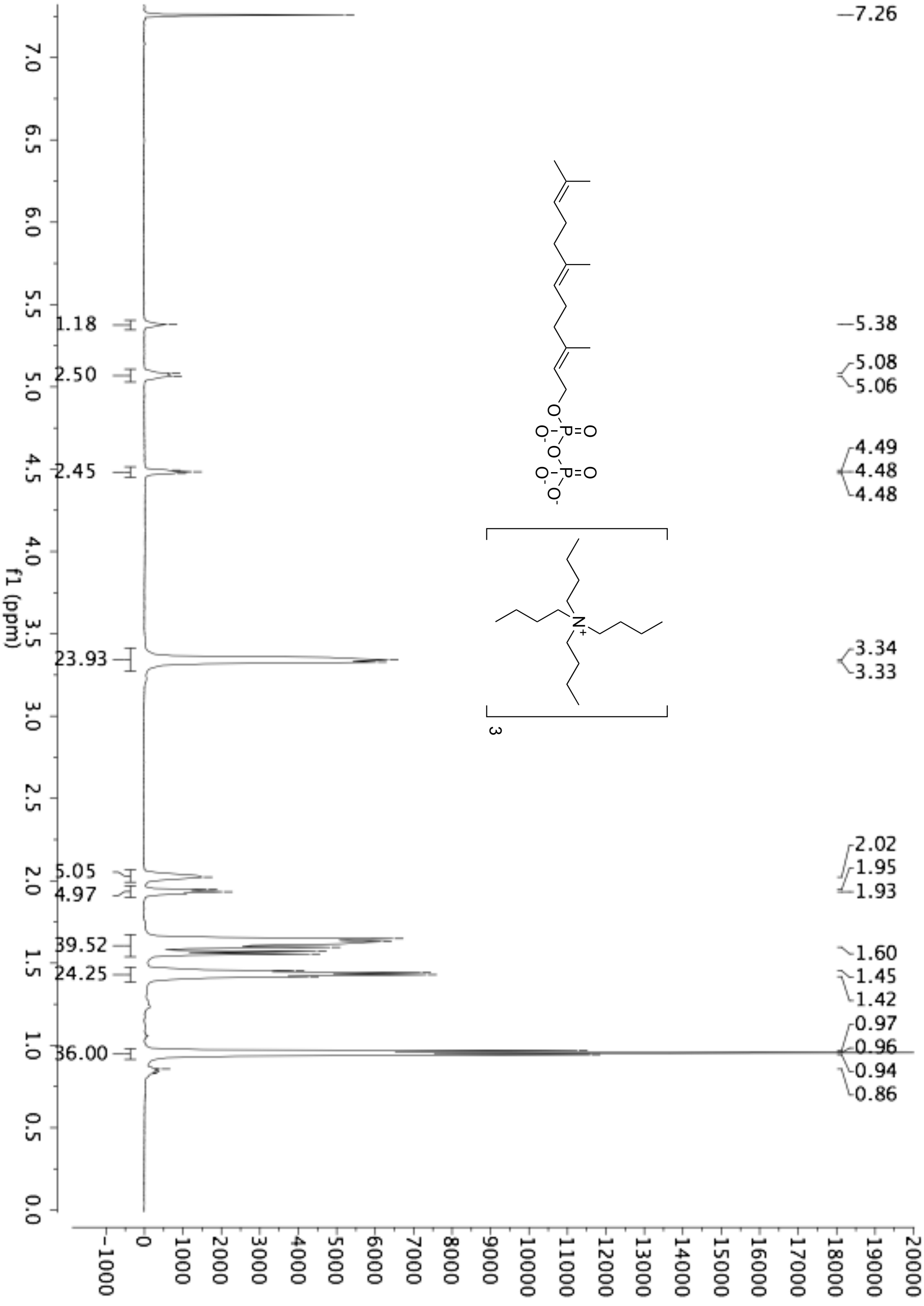

Figure S13. ^{1}H-NMR ($CDCl_3$, 300 MHz) of 12-OH farnesyl chloride (**11**).

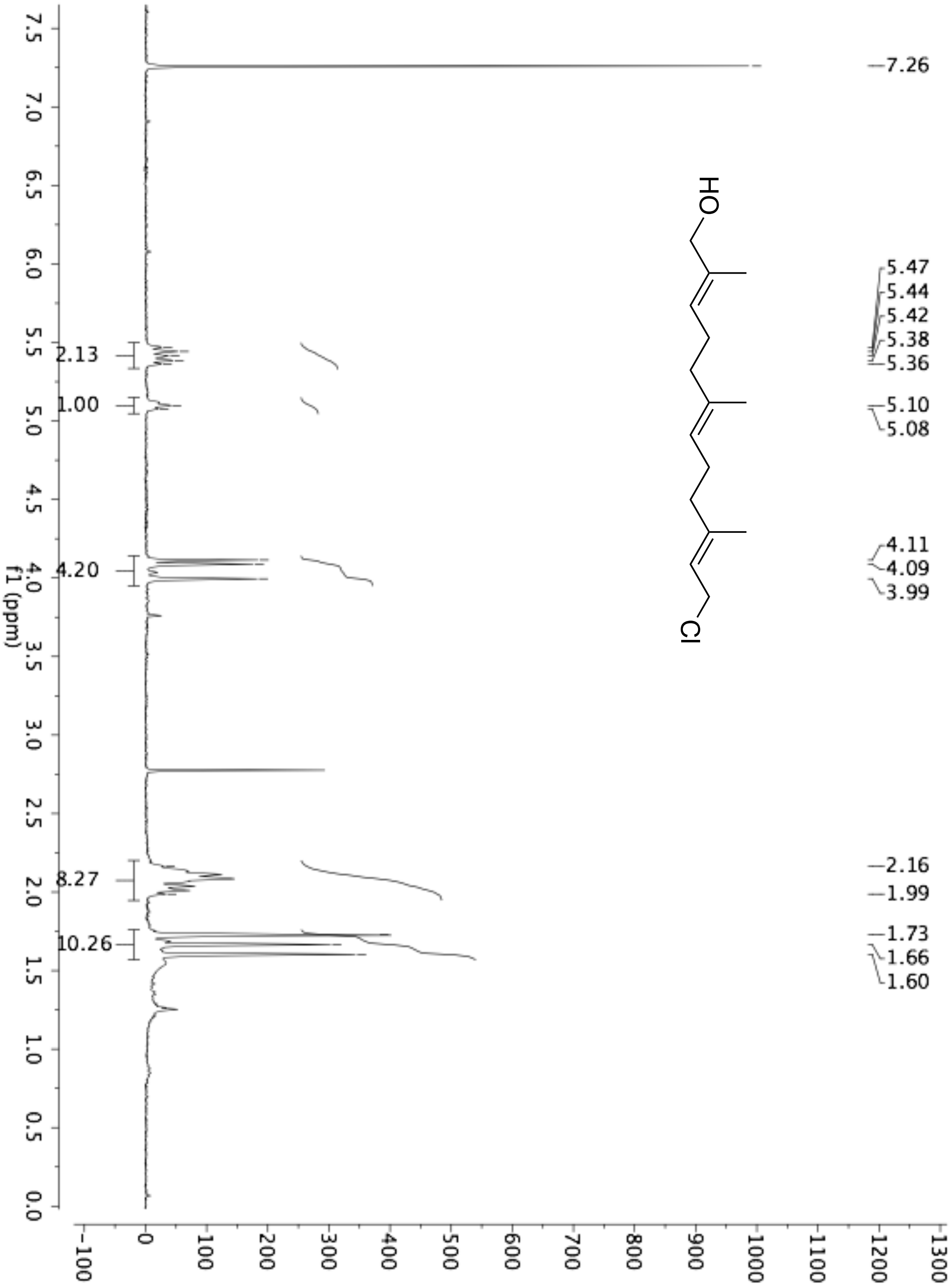

Figure S14. ^{13}C-NMR ($CDCl_3$, 500 MHz) of 12-OH farnesyl diphosphate (**11**).

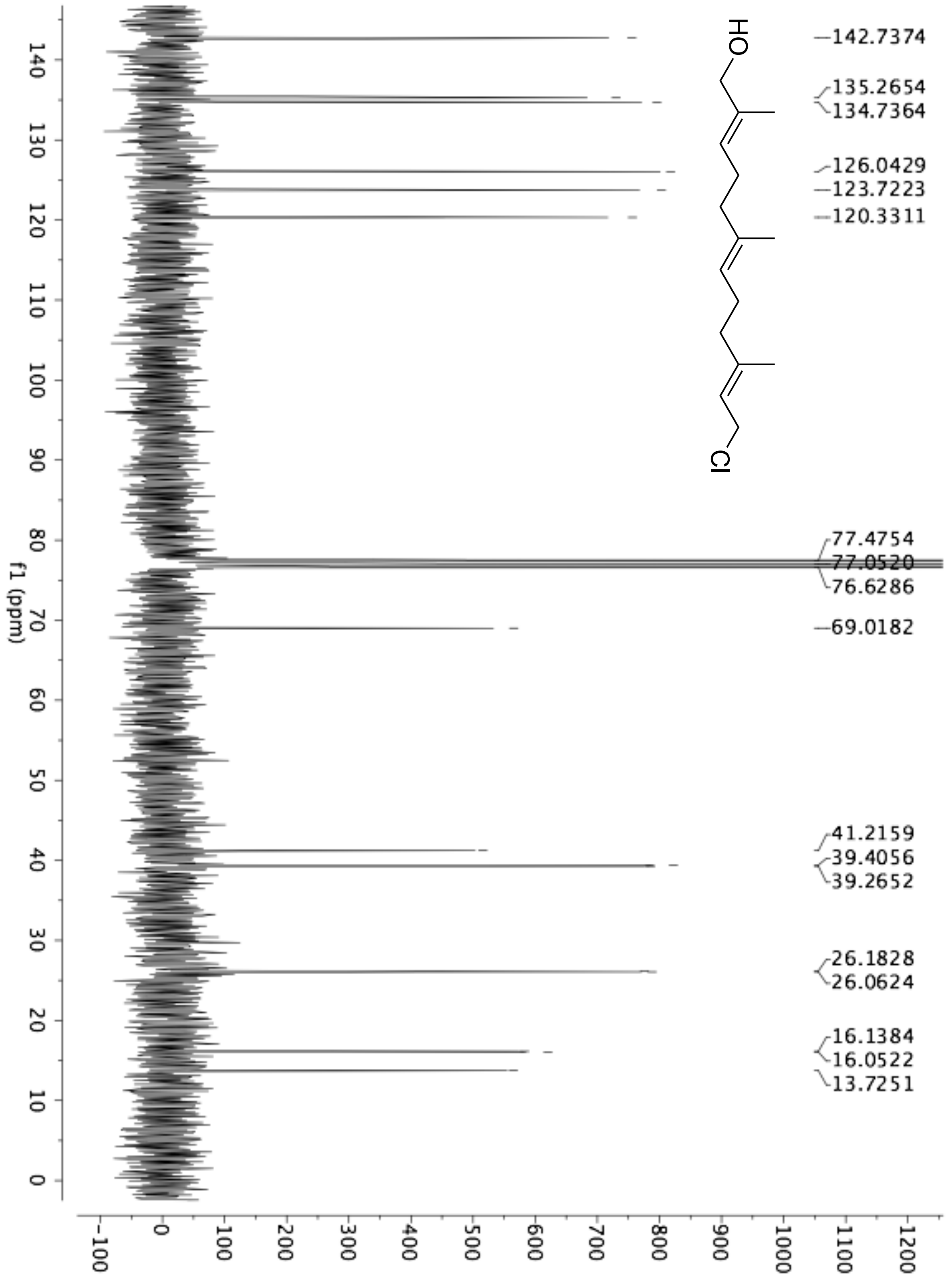

Figure S15. ^{1}H-NMR ($CDCl_3$, 500 MHz) of 12-OH farnesyl diphosphate (**7**).

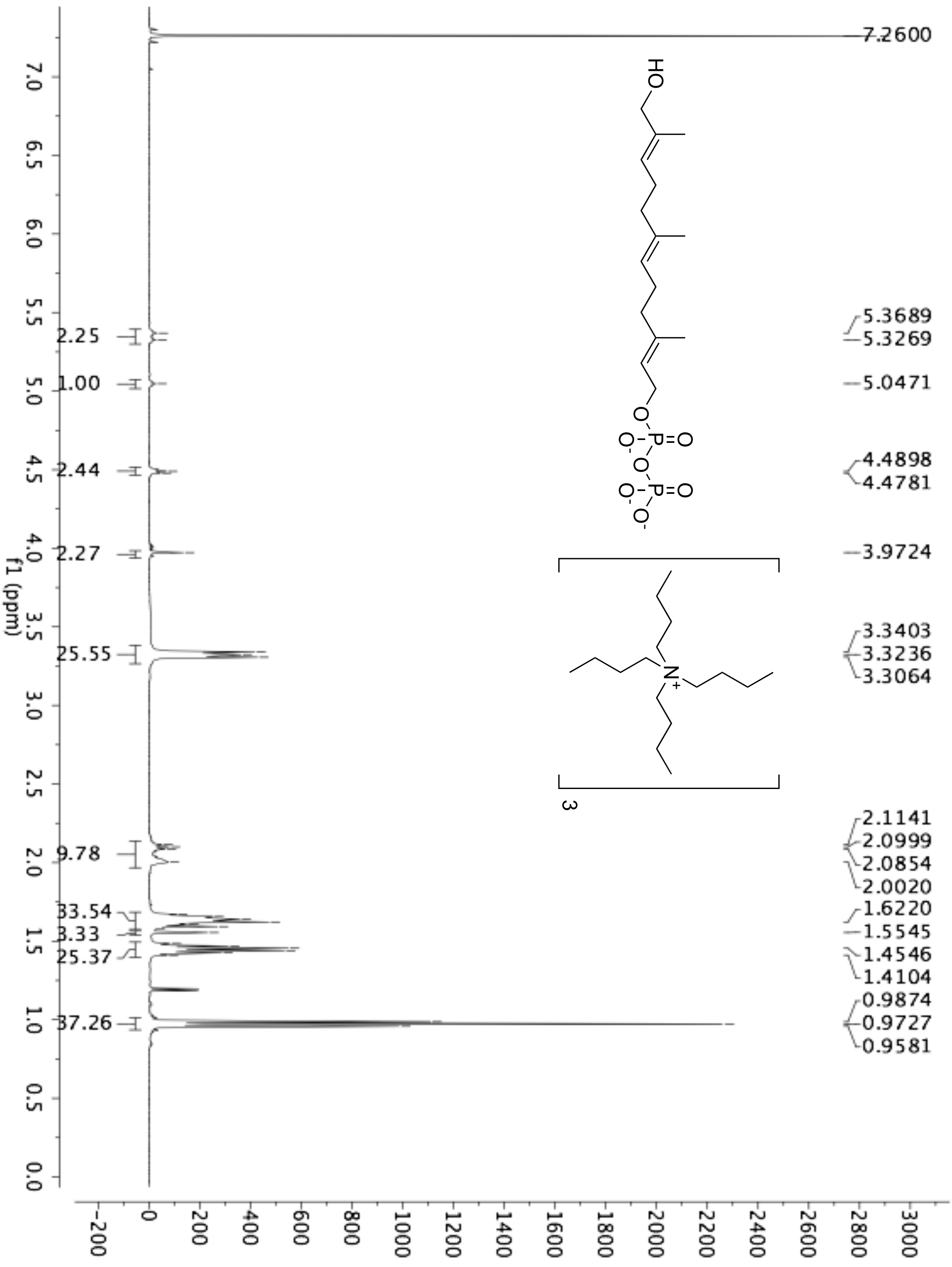

Figure S16. ^{13}C-NMR ($CDCl_3$, 500 MHz) of 12-OH farnesyl diphosphate (**7**).

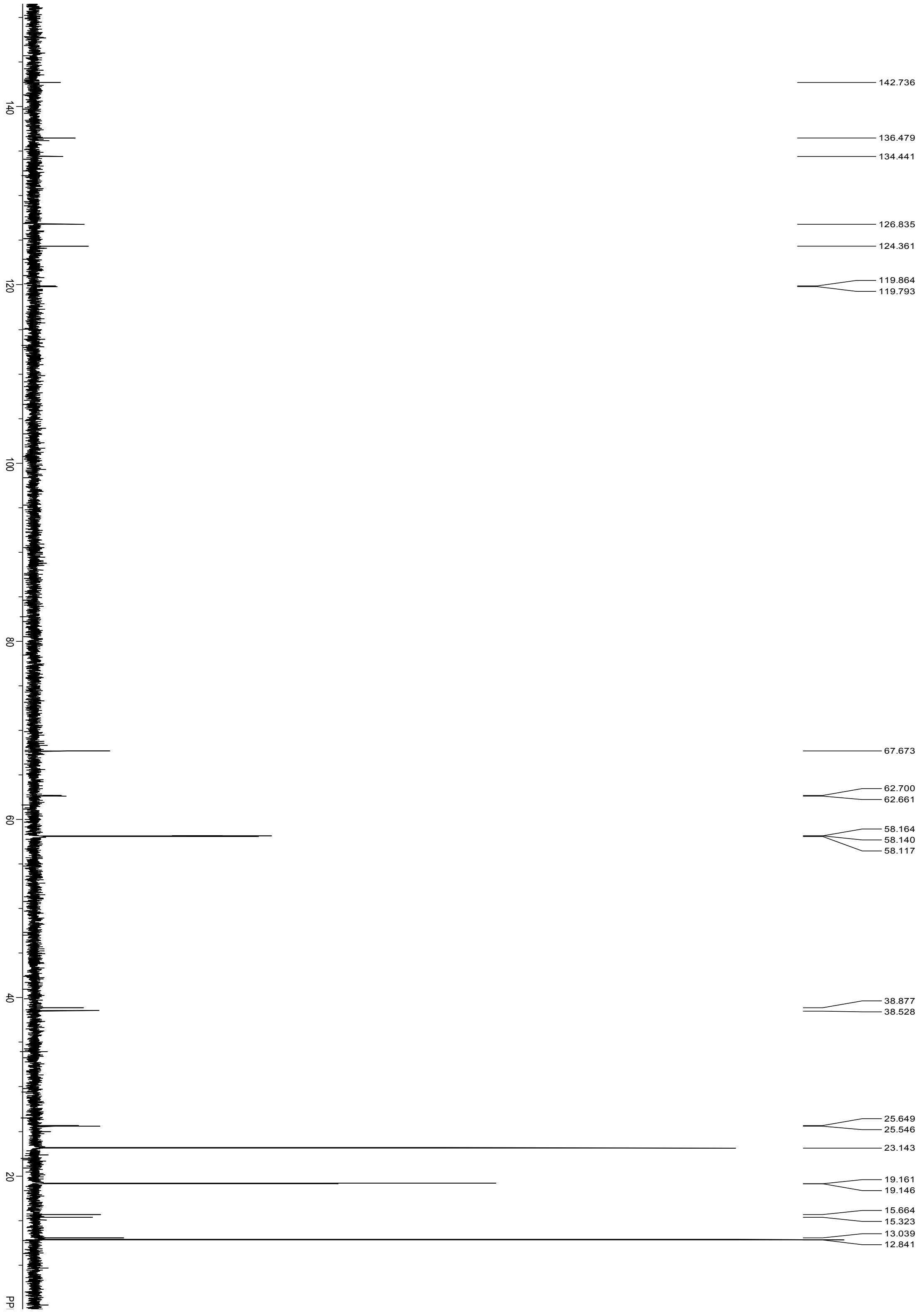

Figure S17. ^{1}H-NMR ($CDCl_3$, 300 MHz) of 12-acetoxyfarnesyl diphosphate (**13**).

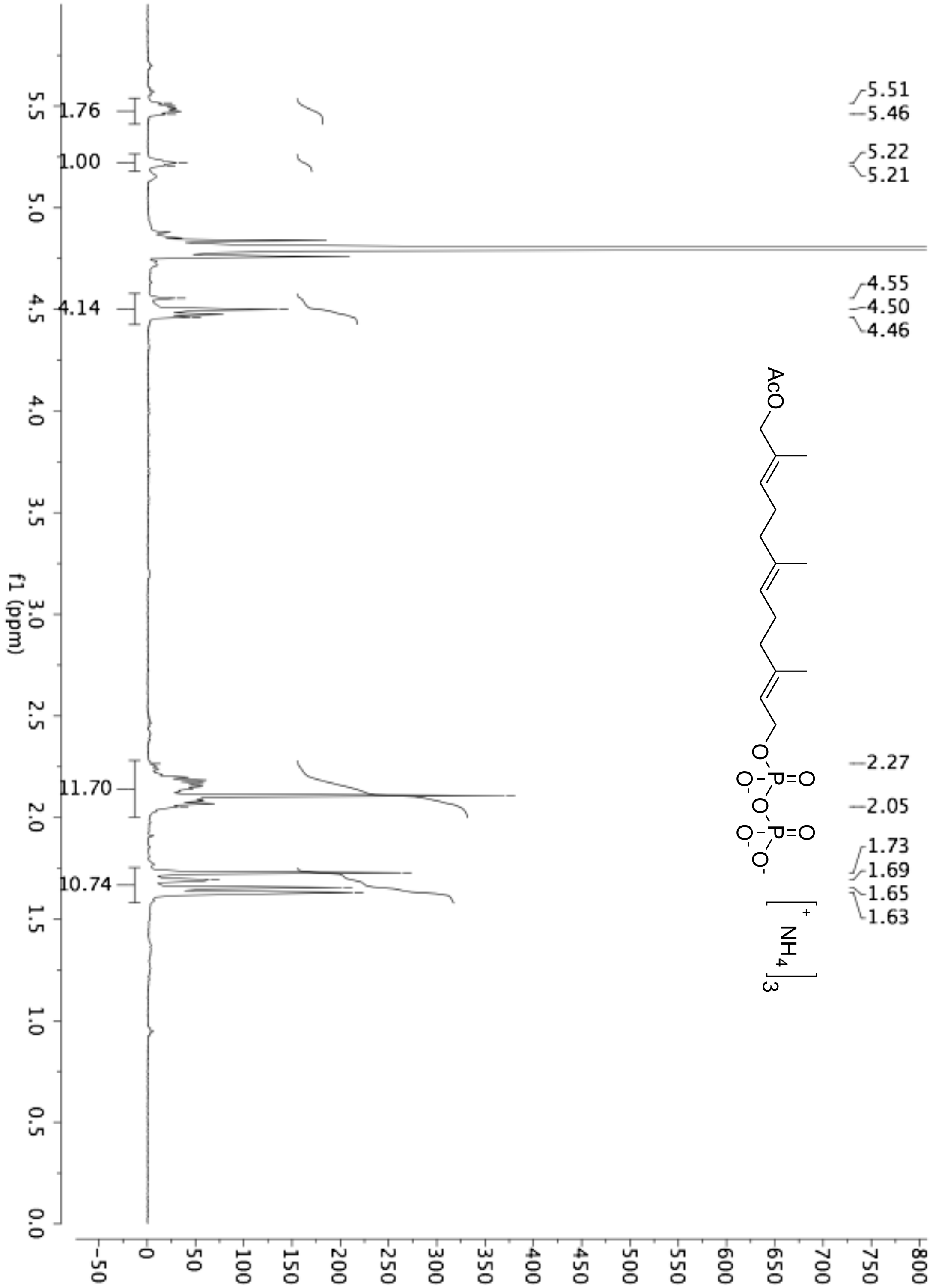

Figure S18. ^{13}C-NMR ($CDCl_3$, 300 MHz) of 12-acetoxyfarnesyl diphosphate (**13**).

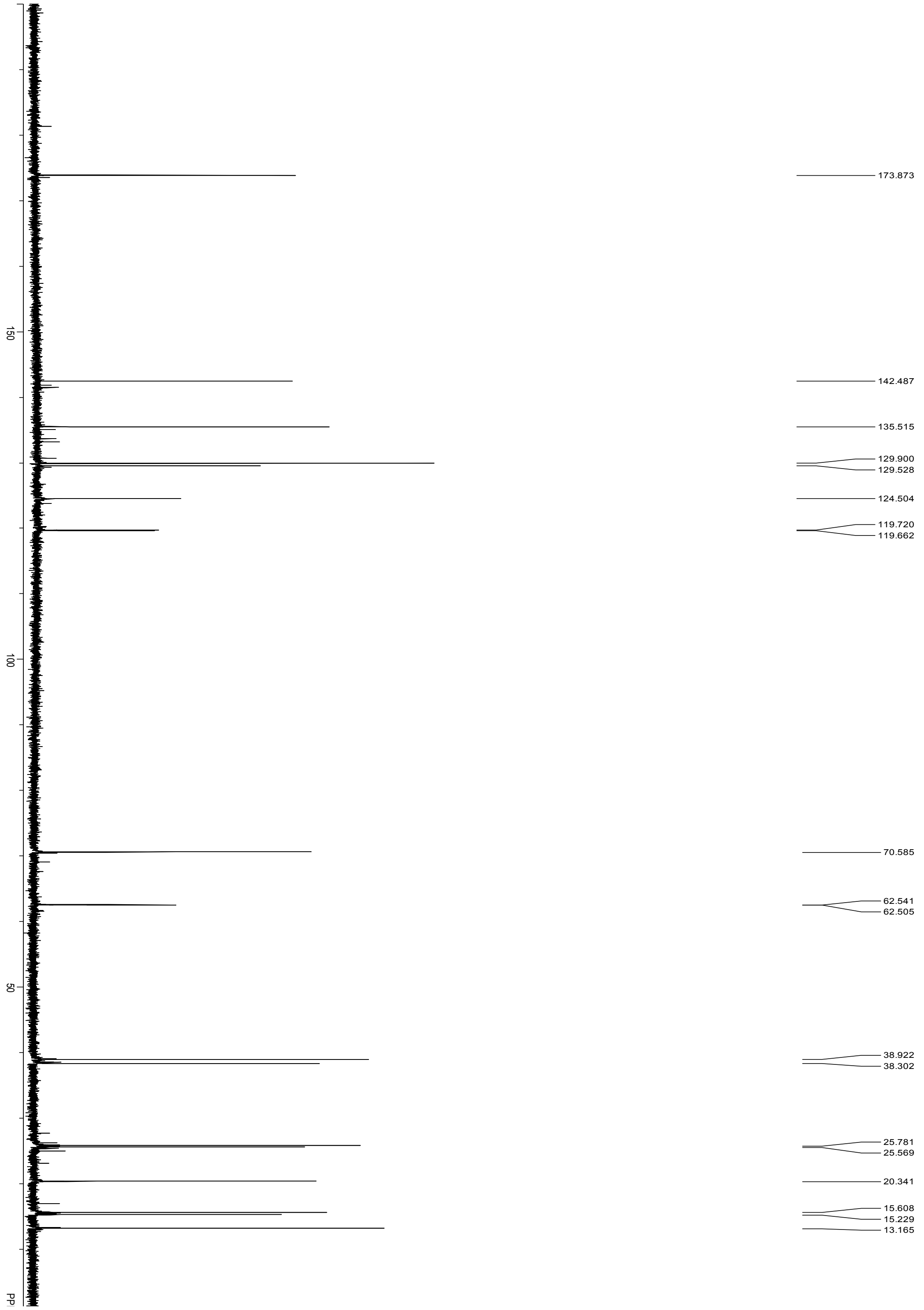

Figure S19. ^{1}H-NMR ($CDCl_3$, 500 MHz) of 13-acetoxyfarnesyl diphosphate (**16**).

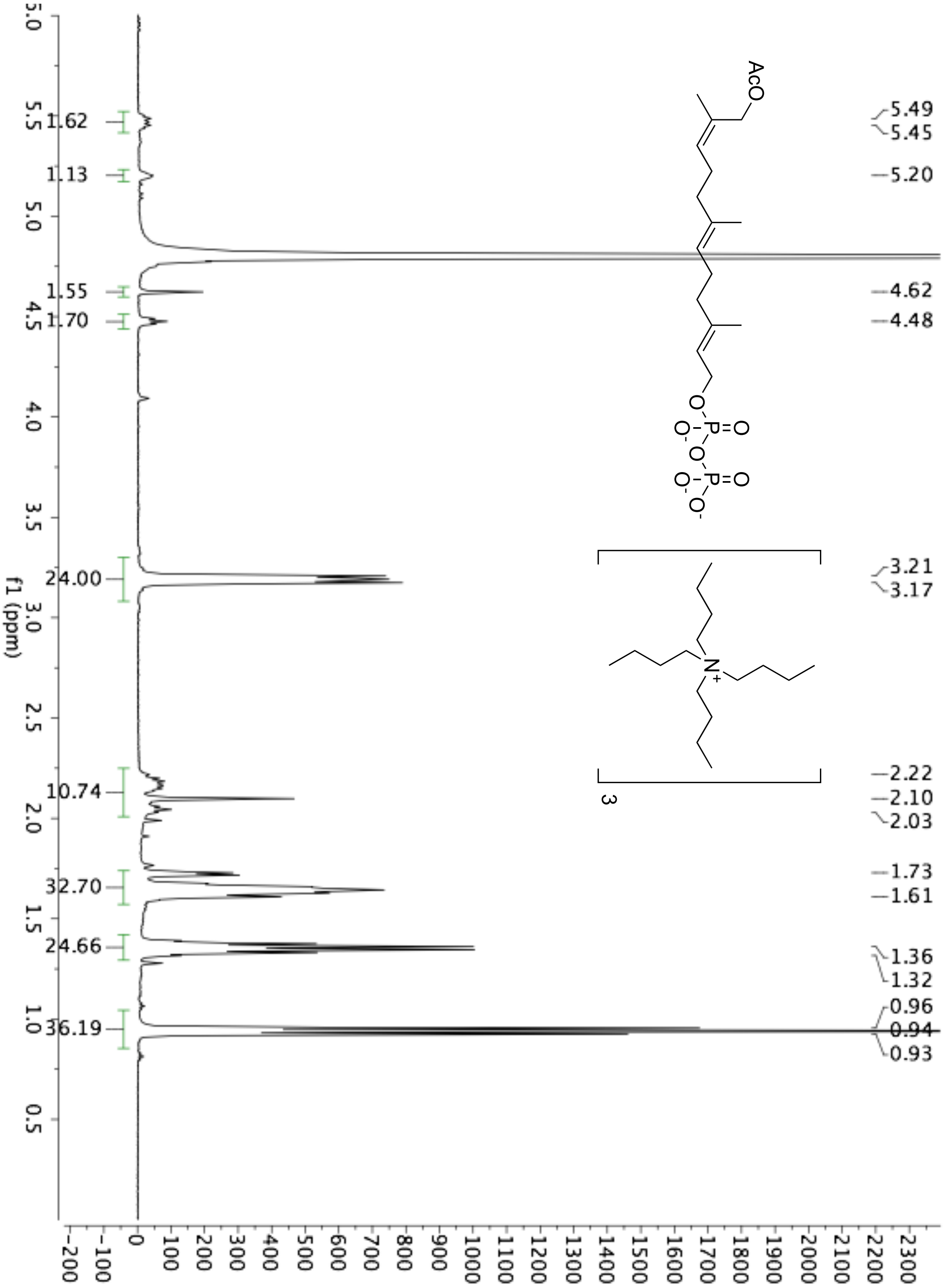

Figure S20. ^{1}H-NMR ($CDCl_3$, 500 MHz) of farnesal.

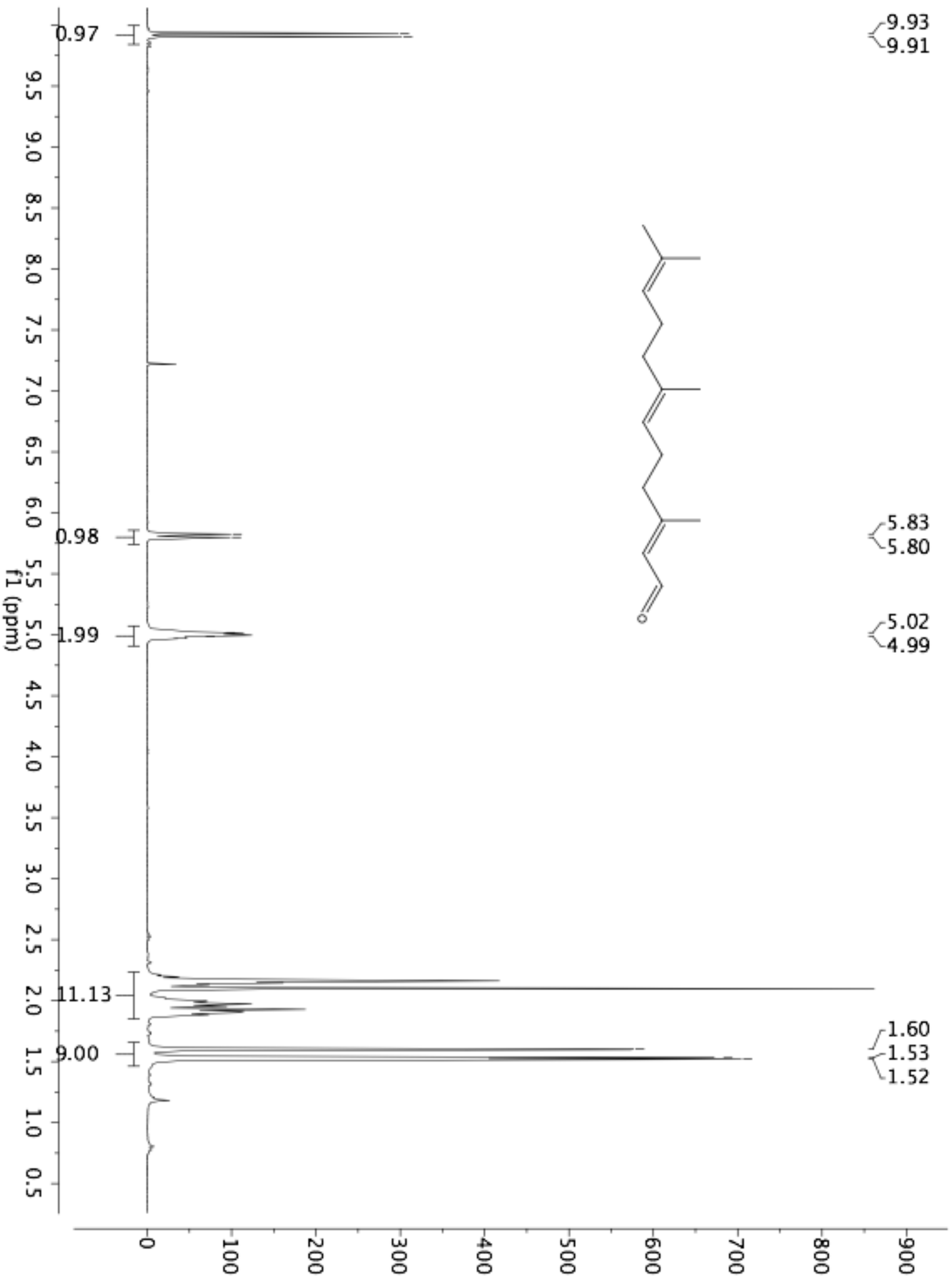

Figure S21. ^{13}C-NMR ($CDCl_3$, 500 MHz) of farnesal.

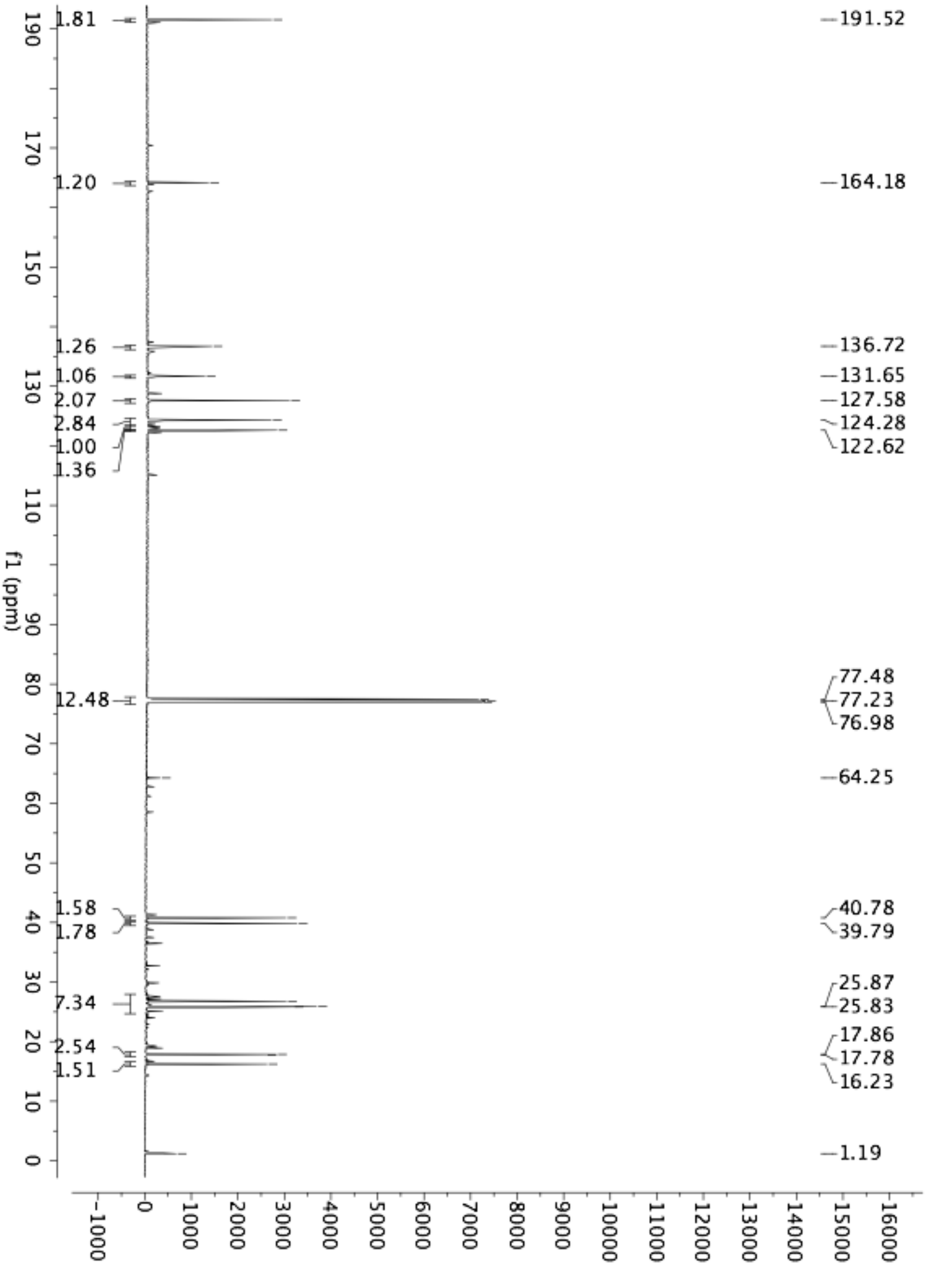

Figure S22. ^{1}H-NMR ($CDCl_3$, 500 MHz) of amorphadiene (**3**).

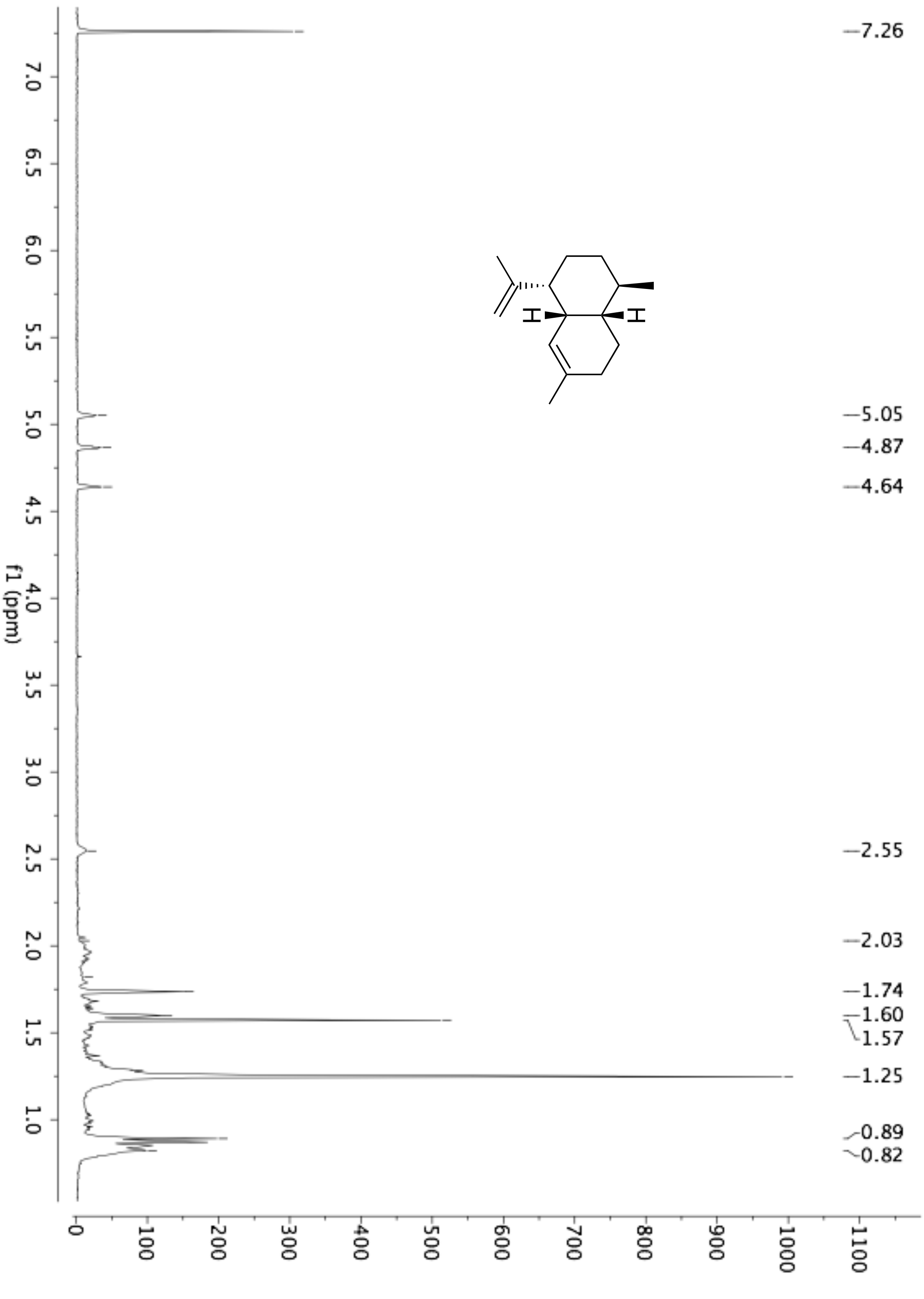

Figure S23. ^{13}C-NMR (CDCl$_3$, 500 MHz) of amorphadiene (**3**).

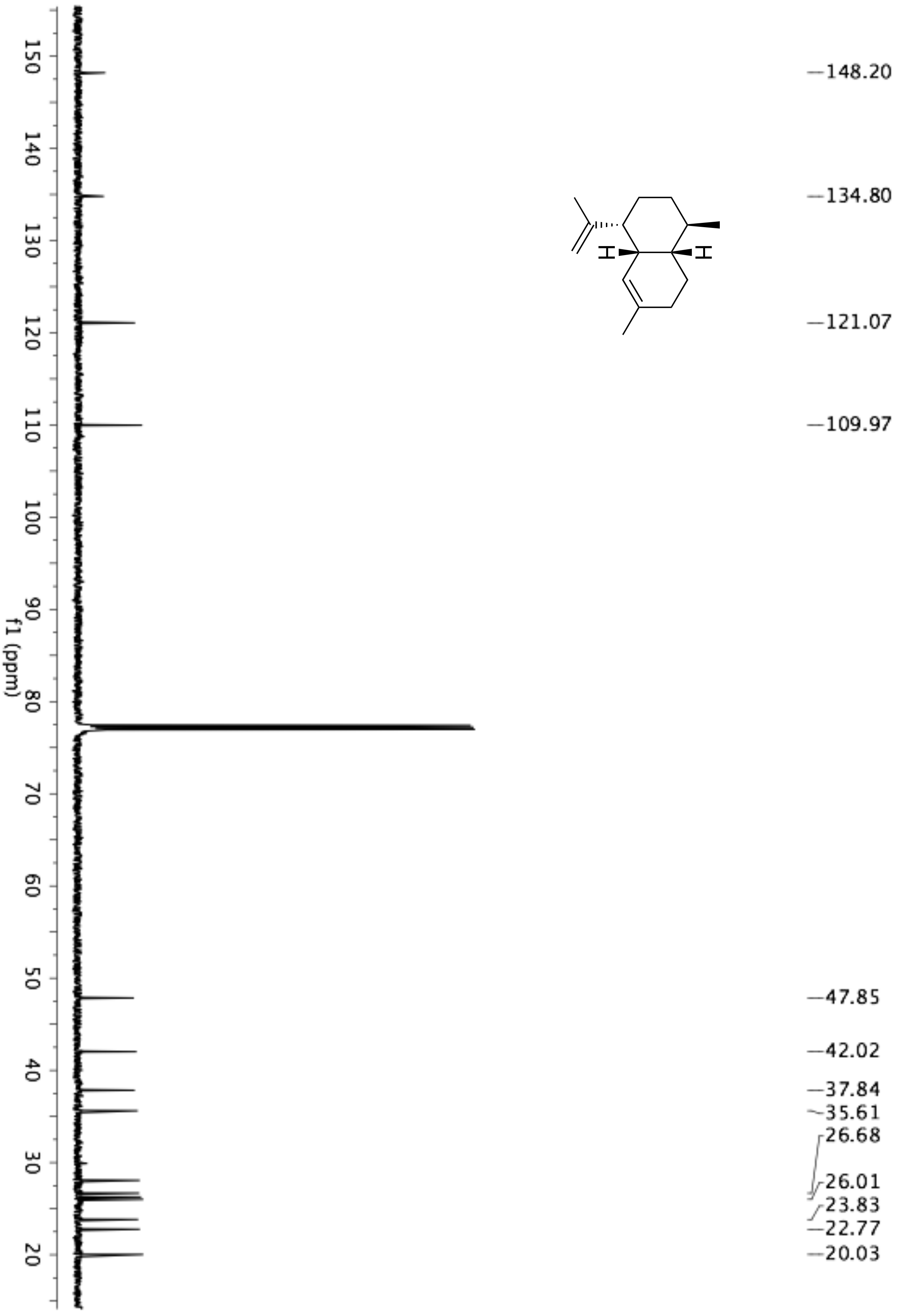

Figure S24. ^{1}H-NMR ($CDCl_3$, 500 MHz) of dihydroartemisinic aldehyde (mixture of epimers) (**4**)

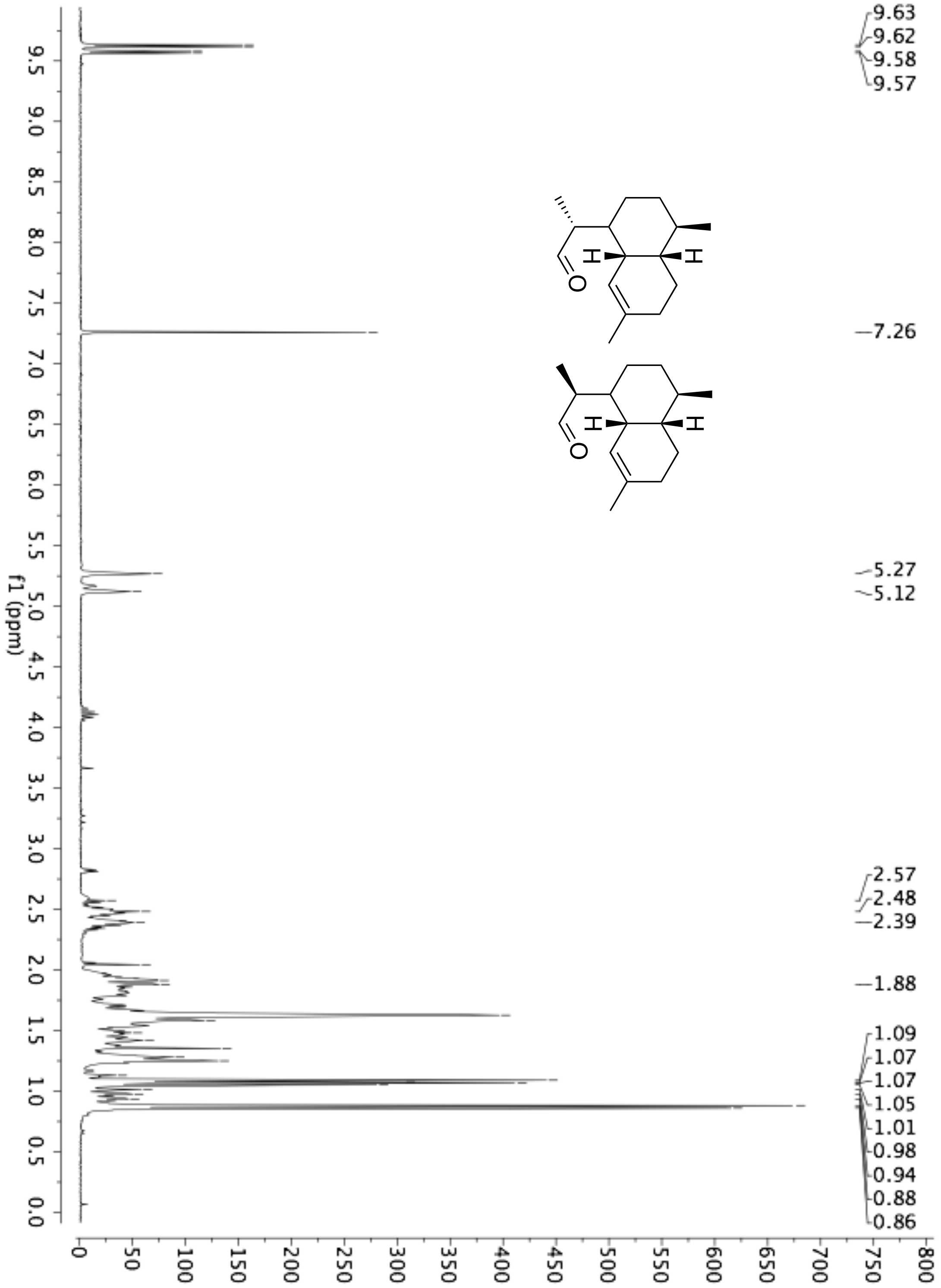

Figure S25. ^{13}C-NMR ($CDCl_3$, 500 MHz) of dihydroartemisinic aldehyde (mixture of epimers) (**4**)

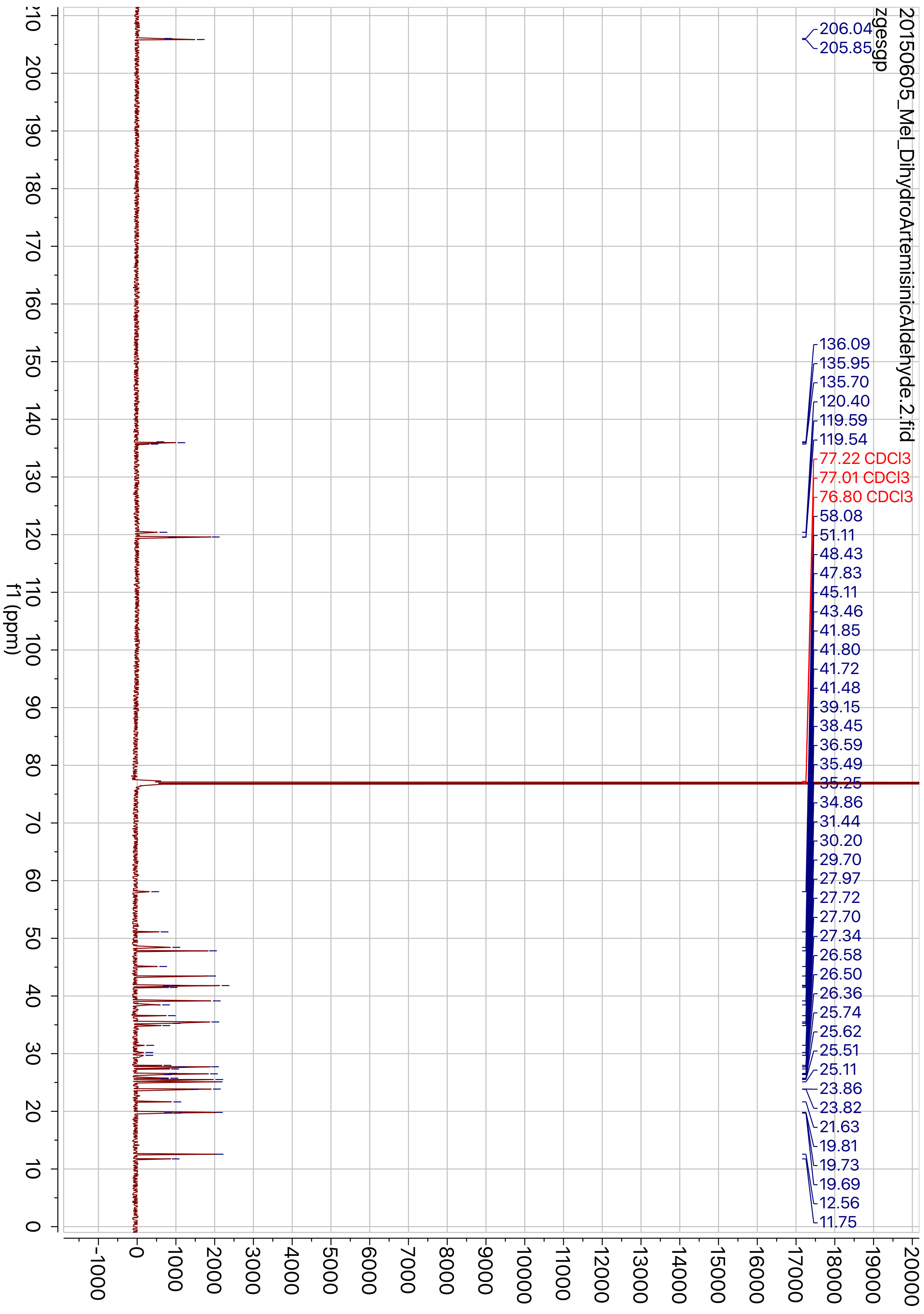

Figure S26. ^{1}H-NMR ($CDCl_3$, 500 MHz) of synthesized (11*R*)-dihydroartemisinic aldehyde (11*R*-**4**).

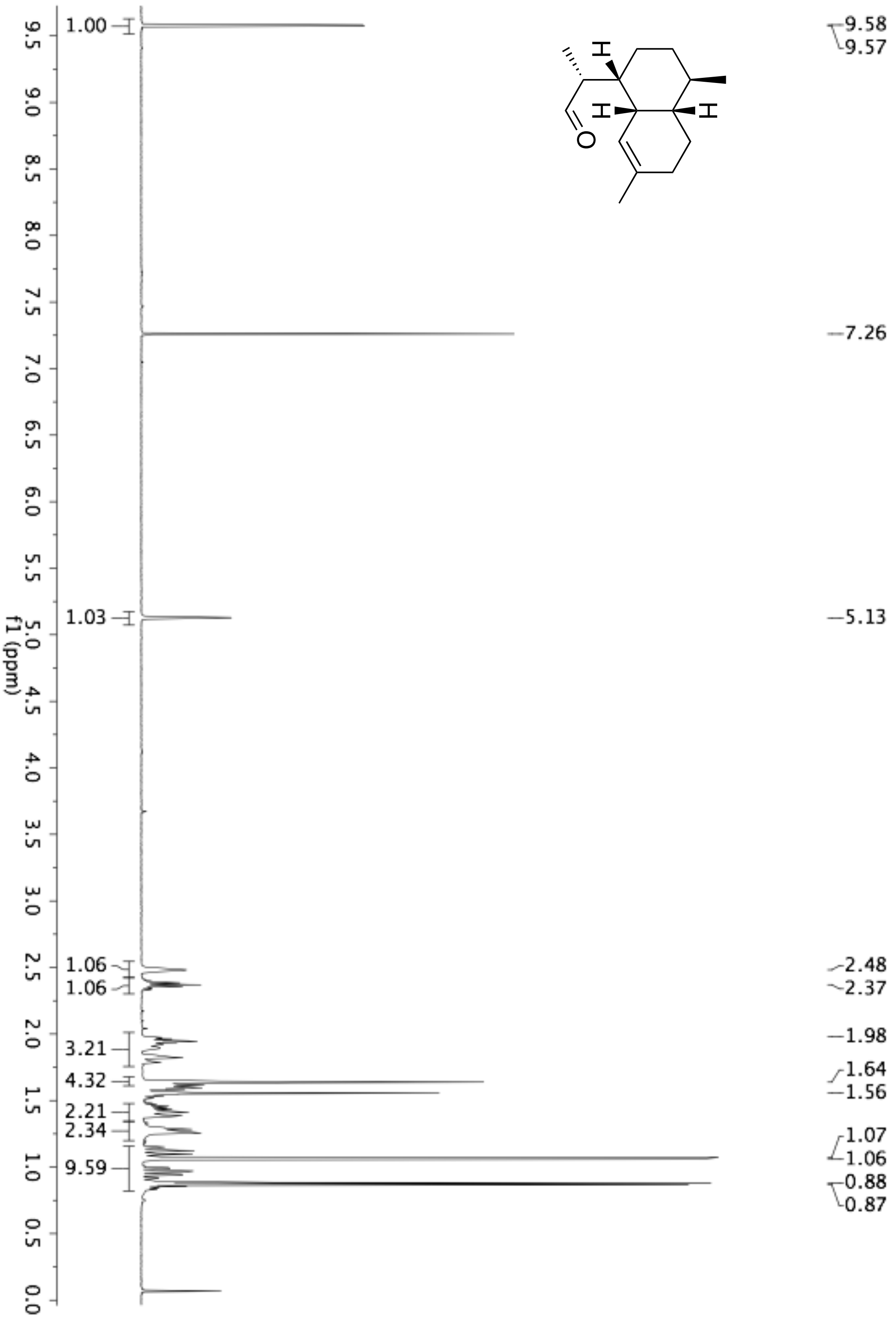

Figure S27. ^{13}C-NMR ($CDCl_3$, 500 MHz) of synthesized (11*R*)-dihydroartemisinic aldehyde (11*R*-**4**).

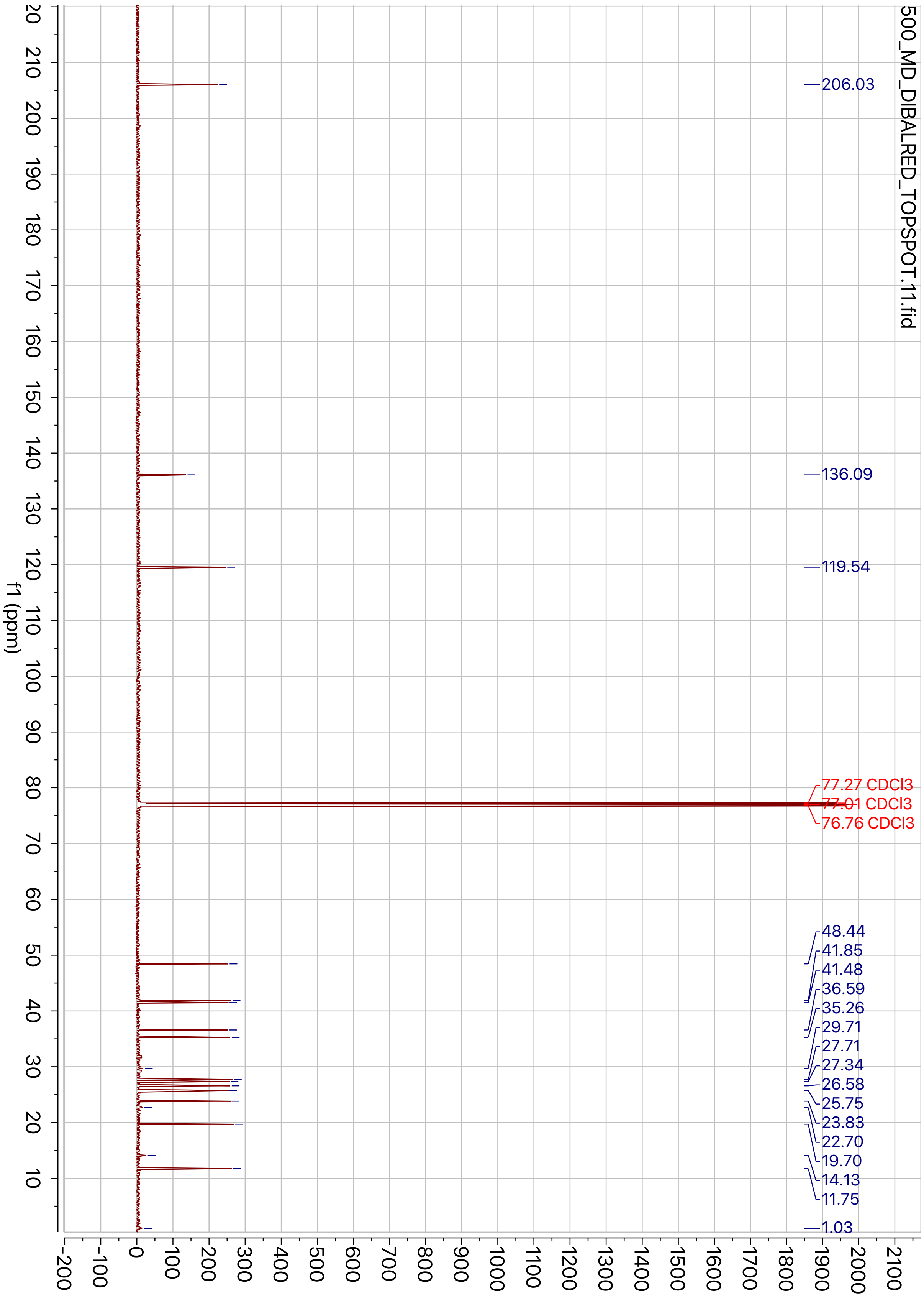

Figure S28. ^{1}H-NMR ($CDCl_3$, 500 MHz) of dihydroartemisinic methyl ester (mixture of epimers) (**18**) after LDA treatment.

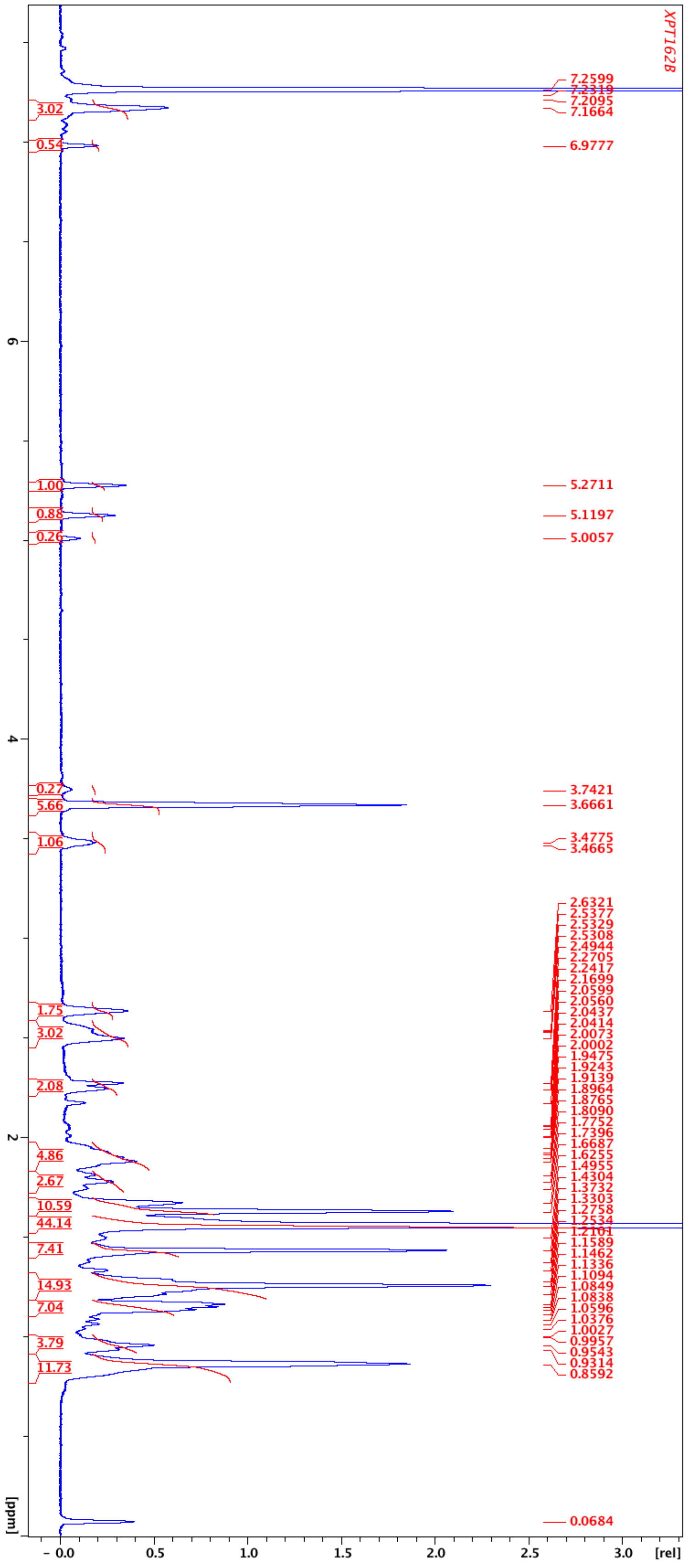

10. References

[1] W. C. Still, M. Kahn, A. Mitra, *J. Org. Chem.* **1978**, *43*, 2923–2925.

[2] V. J. Davisson, A. B. Woodside, T. R. Neal, K. E. Stremler, M. Muehlbacher, C. D. Poulter, *J. Org. Chem.* **1986**, *51*, 4768–4779.

[3] T. Masuda, Y. Odaka, N. Ogawa, K. Nakamoto, H. Kuninaga, *J. Agric. Food Chem.* **2008**, *56*, 597–601.

[4] M. M. Bradford, *Anal. Biochem.* **1976**, *72*, 248–54.

[5] T. Zor, Z. Selinger, *Anal. Biochem.* **1996**, *236*, 302–308.

[6] R. J. Ritchie, T. Prvan, *Biochem. Educ.* **1996**, *24*, 196–206.

[7] H. Lineweaver, D. Burk, *J. Am. Chem. Soc.* **1934**, *56*, 658–666.

[8] K. Johnson, R. Goody, *Biochemistry* **2012**, *50*, 8264–8269.

[9] S. Picaud, L. Olofsson, M. Brodelius, P. E. Brodelius, *Arch. Biochem. Biophys.* **2005**, *436*, 215–226.

References exceeding a limit of 10 authors from the manuscript.

I. A. Graham, K. Besser, S. Blumer, C. A. Branigan, T. Czechowski, L. Elias, I. Guterman, D. Harvey, P. G. Isaac, A. M. Khan, T. R. Larson, Y. Li, T. Pawson, T. Penfield, A. M. Rae,D. A. Rathbone, S. Reid, J. Ross, M. Smallwood, V. Segura, T. Townsend, D. Vyas, T. Winzer, D. Bowles, *Science* **2010**, *327*, 328–331.

P. J. Westfall, D. J. Pitera, J. R. Lenihan, D. Eng, F. X. Woolard, R. Regentin, T. Horning, H. Tsuruta, D. J. Melis, A. Owens, S. Fickes, D. Diola, K. R. Benjamin, J. D. Keasling, M. D. Leavell, D. J. McPhee, N. S. Renninge, J. D. Newman, C. J. Paddon, *PNAS*, **2012**, E111-E118.

C. J. Paddon, P. J. Westfall, D. J. Pitera, K. Benjamin, K. Fisher, D. McPhee, M. D. Leavell, A. Tai, A. Main, D. Eng, D. R. Polichuk, K. H. Teoh, D. W. Reed, T. Treynor, J. Lenihan,H. Jiang, M. Fleck, S. Bajad, G. Dang, D. Dengrove, D. Diola, G. Dorin, K. W. Ellens, S. Fickes, J. Galazzo, S. P. Gaucher, T. Geistlinger, R. Henry, M. Hepp, T. Horning, T. Iqbal, L. Kizer, B. Lieu, D. Melis, N. Moss, R. Regentin, S. Secrest, H. Tsuruta, R. Vazquez, L. F. Westblade, L. Xu, M. Yu, Y. Zhang, L. Zhao, J. Lievense, P. S. Covello, J. D. Keasling, K. K. Reiling, N. S. Renninger, J. D. Newman, *Nature* **2013**, *496*, 528-532.

D. K. Ro, E. M. Paradise, M. Ouellet, K. J. Fisher, K. L. Newman, J. M. Ndungu, K. A. Ho, R. A. Eachus, T. S. Ham, J. Kirby, M. C. Y. Chang, S. T. Withers, Y. Shiba, R. Sarpong, J. D. Keasling, *Nature* **2006**, *440*, 940–943.

C. M. Bertea, J. R. Freije, H. van der Woude, F. W. Verstappen, L. Perk,V. Marquez, J. W. De Kraker, M. A. Posthumus, B. J. Jansen, A. de Groot, M. C. Franssen, H. J. Bouwmeester, *Planta Med.* **2005**, *71*, 40–47.
